# Meta‐analysis of major histocompatibility complex (MHC) class IIA reveals polymorphism and positive selection in many vertebrate species

**DOI:** 10.1111/mec.16726

**Published:** 2022-10-19

**Authors:** Donald C. Dearborn, Sophie Warren, Frank Hailer

**Affiliations:** ^1^ Biology Department Bates College Lewiston Maine USA; ^2^ Roux Institute Northeastern University Portland Maine USA; ^3^ Organisms and Environment, School of Biosciences Cardiff University Cardiff UK; ^4^ Present address: Department of Health Policy London School of Economics and Political Science London UK

**Keywords:** allelic diversity, diversifying selection, heterodimer, meta‐analysis, MHC, pathogen‐mediated selection

## Abstract

Pathogen‐mediated selection and sexual selection are important drivers of evolution. Both processes are known to target genes of the major histocompatibility complex (MHC), a gene family encoding cell‐surface proteins that display pathogen peptides to the immune system. The MHC is also a model for understanding processes such as gene duplication and trans‐species allele sharing. The class II MHC protein is a heterodimer whose peptide‐binding groove is encoded by an MHC‐IIA gene and an MHC‐IIB gene. However, our literature review found that class II MHC papers on infectious disease or sexual selection included IIA data only 18% and 9% of the time, respectively. To assess whether greater emphasis on MHC‐IIA is warranted, we analysed MHC‐IIA sequence data from 50 species of vertebrates (fish, amphibians, birds, mammals) to test for polymorphism and positive selection. We found that the number of MHC‐IIA alleles within a species was often high, and covaried with sample size and number of MHC‐IIA genes assayed. While MHC‐IIA variability tended to be lower than that of MHC‐IIB, the difference was only ~25%, with ~3 fewer IIA alleles than IIB. Furthermore, the unexpectedly high MHC‐IIA variability showed clear signatures of positive selection in most species, and positive selection on MHC‐IIA was stronger in fish than in other surveyed vertebrate groups. Our findings underscore that MHC‐IIA can be an important target of selection. Future studies should therefore expand the characterization of MHC‐IIA at both allelic and genomic scales, and incorporate MHC‐IIA into models of fitness consequences of MHC variation.

## 1 INTRODUCTION

Across jawed vertebrates, the genes of the major histocompatibility complex (MHC) make cell‐surface proteins that display pathogen peptides to the immune system (Murphy & Weaver, 2017). The MHC is central to host‐pathogen interactions, and different MHC alleles encode proteins that can differ in their effectiveness against particular pathogens (Sepil et al., 2013; Wegner et al., 2003). This makes MHC variation a key component of host–parasite dynamics (Jeffery & Bangham, 2000; Spurgin & Richardson, 2010). In turn, fitness differences in MHC genotypes among prospective mates and future offspring can make MHC an important target of sexual selection (Kamiya et al., 2014; Milinski, 2006). More broadly, studying the MHC has also become valuable for understanding an array of evolutionary processes, including frequency‐dependent selection (Phillips et al., 2018), heterozygote advantage (Penn et al., 2002), gene duplication and deletion (Nei & Rooney, 2005), copy number variation (Minias et al., 2019), gene conversion (Spurgin et al., 2011), adaptive introgression (Dudek et al., 2019), and trans‐species polymorphism (Lighten et al., 2017). The MHC is therefore a key focus of a range of studies across evolution and ecology.

**FIGURE 1:**
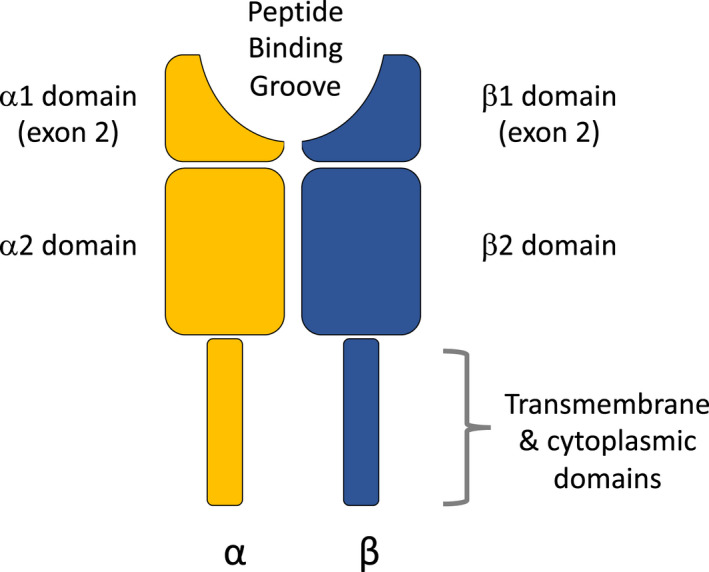
Schematic illustration of the class II MHC protein. This protein is a heterodimer, in which both the alpha subunit (left; encoded by MHC‐IIA loci) and beta subunit (right; encoded by MHC‐IIB loci) contribute to the peptide binding groove

MHC proteins fall in two categories: class I MHC is expressed on all cell types and displays intracellularly derived peptides, whereas class II MHC is only on professional antigen‐presenting cells and displays peptides from extracellularly encountered pathogens (Murphy & Weaver, 2017). Structurally, the peptide binding groove of the MHC protein is encoded by a single gene in class I MHC, whereas two different genes jointly encode the binding groove in class II MHC (Figure 1). Specifically, class II MHC is a heterodimer consisting of an alpha subunit from an MHC‐IIA gene, and a beta subunit from an MHC‐IIB gene. Both of the class II subunits have amino acids that directly interact with the pathogen peptide (Brown et al., 1993) and thus have the potential to influence the effectiveness or specificity of peptide binding and pathogen defence.

Across taxa, though, “…most research has focused on the second and third exons of class I genes and the second exon of class II B genes, because of their traditional consideration as primary targets of pathogen‐mediated selection” (Canal et al., 2010). A large body of single‐species studies has found that MHC class I and class IIB routinely exhibit high levels of allelic polymorphism (i.e., genetic variability at a given locus) within and between populations and a history of positive selection at peptide binding sites (Höglund, 2009; Piertney & Oliver, 2006; Radwan et al., 2020). Perhaps as a result, class II meta‐analyses or review papers typically focus on MHC‐IIB (Bernatchez & Landry, 2003; Burri et al., 2014; Hess & Edwards, 2002; Minias et al., 2018, 2019; Winternitz et al., 2013) In contrast, MHC‐IIA loci appear to have received comparatively little attention, despite their contribution to the MHC protein's peptide binding groove (Figure 1) and hence their potential importance for understanding fitness consequences of MHC variation.

Much of the early studies on MHC‐IIA focused on mammals, which led to ‐ and then was further facilitated by ‐ the existence of widely used sets of PCR primers and a clearer understanding of orthology and genomic organization than in other taxa (Kumánovics et al., 2003; Maccari et al., 2017; Trowsdale & Knight, 2013). This early work found evidence of polymorphism in the DQA lineage of MHC‐IIA in a range of mammals (Bontrop et al., 1999; Gyllensten & Erlich, 1989; Janova et al., 2009; Scott et al., 1991; Wagner et al., 1996). More recently, a multispecies analysis of mammalian MHC‐DQ genes suggested that MHC‐IIA might indeed be an important target of diversifying selection: data from the DQA lineage in 10 nonhuman mammal species showed substantial evidence of positive selection within species (Amills et al., 2008), highlighting the potential for MHC‐IIA to warrant greater attention in studies of MHC ecology and evolution. In the 15 years since that multispecies analyses of Amills et al. (2008), MHC‐IIA sequencing efforts have expanded both within and beyond mammals in a way that makes this a valuable moment to assess the state of the field.

Our synthesis has two main aims. First, we assess the functional attention paid to MHC‐IIA by examining the extent to which MHC‐IIA is underrepresented, relative to MHC‐IIB, in studies of sexual selection or disease association. Second, we compile sequences and associated metadata on MHC‐IIA genes in nonhuman vertebrate taxa to test for two phenomena that are widely understood as fundamental to the evolution of MHC‐IIB: (1) Does MHC‐IIA show pronounced genetic variability (allelic polymorphism) within species? and (2) Is the existing variation in MHC‐IIA consistent with a process of positive selection rather than neutral evolution and purifying selection? We address these questions by analysing existing MHC‐IIA sequence data in a standardized way across a variety of taxa, including a test of three predictor variables (sample size, number of genes assayed, and taxonomic group within the vertebrates) that could contribute to differences in observed levels of MHC‐IIA polymorphism or selection regime in different species. We also make direct, paired comparisons of the polymorphism and selection regime of MHC‐IIA and MHC‐IIB genes, for cases where population‐matched data for both classes of loci are available.

## 2 MATERIALS AND METHODS

### 2.1 Prevalence of MHC‐IIA versus MHC‐IIB in studies of sexual selection or infectious disease

We began by assessing the extent to which studies of sexual selection or infectious disease associations in class II MHC might be disproportionately focused on MHC‐IIB rather than MHC‐IIA. To that end, we conducted literature searches spanning the years 2000 to 2021 for nonhuman empirical studies of those topics, and we then categorized those studies based on whether they contained data on MHC‐IIA, MHC‐IIB, or both.

Specifically, to assess the sexual selection literature we searched Scopus in November 2021 for papers whose title, abstract, or keywords included (mhc OR histocompatibility) AND (“mate choice” OR “mating” OR “sexual select*”) AND (class AND II). This yielded 354 papers published after 1999. We then excluded review papers, papers about humans, papers that were not actually about sexual selection or mate choice, and papers that lacked class II MHC sequence or haplotype data. We also excluded six studies (4 horse, 2 Soay sheep) that used multigene haplotypes but did not analyse or interpret data at the gene level. Our full set of criteria yielded 116 papers, as listed in Section 1 of the Supporting Information.

To assess the literature on infectious disease associations with MHC‐IIA or MHC‐IIB alleles, we searched Scopus in November 2021 for papers whose title, abstract, or key words included (mhc OR histocompatibility) AND (“disease*” OR “pathogen*” OR “parasite*” OR “infect*”) AND (“class II” OR “class‐II”). This yielded 655 papers published after 1999. We then excluded review papers, papers about humans, papers about laboratory animal models of human disease, papers on noninfectious disease, and papers that lacked class II MHC sequence or haplotype data. We also excluded 12 papers (7 chicken, 4 mouse, 1 *Xenopus*) that knew or assessed multigene MHC haplotypes spanning at least one MHC‐IIA gene and one MHC‐IIB gene but that did not consider MHC‐IIA or MHC‐IIB specifically, nor did they attempt to ascertain which genes within the haplotypes were actually the source of the association between haplotype and disease state. Our full set of criteria yielded 95 papers, as listed in Section 1 of the Supporting Information.

We then assessed whether MHC‐IIA was becoming more prevalent in such studies over time. To test whether MHC‐IIA data was more likely to appear in more recent papers on these topics, we conducted a logistic regression analysis for the 116 mate choice papers and, separately, for the 95 disease association papers. The presence or absence of MHC‐IIA data in a class II MHC paper was modelled as a binomial GLM with a logit link, with Year of publication as a continuous predictor variable:

$${\text{IncludesIIA}}_{i}\;∼\;\text{Binomial}\;\left(\pi_{i},n_{i}\right)$$

$$E\left({\text{IncludesIIA}}_{i}\right)\;\sim \;n_{i}\;\;x\;\;\pi_{i}\;\;\text{and}\;\;\mathrm{var}\left({\text{IncludesIIA}}_{i}\right)\;=\;n_{i}\;\;x\;\;\pi_{i}\;\;x\;\;\left(1–\pi_{i}\right)$$

$$\text{logit}\;\left(\pi_{i}\right)=\eta_{i}$$

$$\eta_{i}=\beta_{1}+\beta_{2}\;x\;{\text{Year}}_{i}$$

### 2.2 MHC‐IIA diversity and selection: Literature search

We surveyed the literature for studies of MHC‐IIA polymorphism, initially by searching in Scopus for (MHC OR “major histocompatibility complex”) AND (“class II alpha" OR “class II α” OR “class II A"), and then scanning those papers' references and papers that cited them, expanding forward until no new papers were detected. Papers were used in the analysis if they met the following inclusion criteria:

- Accessible sequence data of MHC‐IIA alleles. If data were not available in a repository (e.g., GenBank or IPD‐MHC [Maccari et al., 2017]) or in the paper itself, we emailed authors to request data.
- Sequences that spanned at least 90% of exon 2, the exon which encodes the alpha 1 (MHC‐IIA) or beta 1 domain (MHC‐IIB) of the protein.
- A defined sample size of at least two individuals.
- Samples drawn from a biologically defined group. This was typically wild‐caught individuals from one or several populations, but alternatively could include animals from aquaculture or other farmed settings, fish bought at a specific market, or domesticated animals of particular breeds. We excluded a few studies of captive or domesticated animals where the sampled individuals were deliberately chosen for characterization based on a priori knowledge of their MHC genotypes (e.g., using only known MHC‐homozygotes).
- One data set per species. This criterion came into play in 11 species for which there were multiple papers that met the other inclusion criteria. In those cases, we selected the paper that provided the most thorough characterization of MHC polymorphism (based on sample size, breadth of sampling, number of genes, and extent of exon 2 coverage). There were no cases in which data on different loci in a species were combined across studies, mainly because of differences between studies in which populations were sampled.

Because MHC characterization methodology varies so widely (O'Connor et al., 2019), we did not restrict our set of studies based on other aspects of methodology, including the number of genes that the authors estimated that they were amplifying, the number of primer pairs used to generate the sequences, the use of DNA versus RNA source material, or the sequencing or sequence characterization methodology (e.g., SSCP, Illumina, cloning and Sanger).

For papers that met our inclusion criteria for MHC‐IIA data, we then searched for MHC‐IIB data (in the same paper or in a different paper) from the same species. Here we applied the same inclusion criteria, with the additional requirement that the MHC‐IIB data be drawn from the same population as the MHC‐IIA data, to allow an apples‐to‐apples paired comparison.

We gave careful consideration to issues involving the number and orthology of genes. Studies of some well‐characterized mammalian systems are able to distinguish between orthologues (e.g., DRA, DQA, DPA). However, most studies of nonmodel systems lack information on the existence of orthology or copy number, and lack locus‐specific primers. In fact, many studies do not know the number of genes that exist in their organism, nor the number of genes or gene copies that are being amplified by their PCR primers. Instead, the minimum number of amplified genes is inferred based on the maximum number of sequences recovered from one individual. Consequently, studies of nonmodel species routinely produce a set of MHC‐IIA sequences that are from multiple genes and that cannot be sorted into genes or lineages. As a result, any analysis of genetic variability or selection history must jointly examine MHC‐IIA alleles irrespective of gene lineage. For consistency, we adopted the same approach for the subset of species for which gene lineages are well characterized (typically in mammals). That is, if a study separately amplified DQA and DRA, we pooled all alleles into a single characterization of polymorphism and selection. This is consistent with a functional allelic framework across loci (rather than a gene‐centred framework) to thinking about how much MHC variation exists and how selection has shaped that variation over time. Further exploration of this topic with respect to the data sets in this analysis is provided in Section 2 of the Supporting Information.

### 2.3 MHC‐IIA diversity and selection: Sequence alignment within species

For highly similar sequences, we used Sequencher 5.4 (Gene Codes Corporation, Ann Arbor, MI, USA) to separately align each species' MHC‐IIA or MHC‐IIB alleles, or used the original authors' alignment when available. For more divergent sequences within species, we made amino acid alignments using Muscle in MEGA X (Kumar et al., 2018). After aligning a species' MHC‐IIA or MHC‐IIB sequences, we verified or trimmed the sequences to exon 2, trimmed the initial base(s) and final base(s) to include only complete codons, and eliminated any alleles that had nonunique nucleotide sequences after trimming.

### 2.4 Allelic diversity in MHC‐IIA

We imported the aligned sequences of each species separately into MEGA X to measure intraspecific polymorphism in MHC‐IIA. We measured each species' variability in nucleotide sequences by counting the number of unique alleles and by computing the average p‐distance between sequences using pairwise deletion. Species were included in polymorphism analyses even if they had only a single allele, in which case DNA and amino acid allele numbers were set to one, and nucleotide/amino acid p‐distances were recorded as zero. Nearly all MHC polymorphism studies lacked allele frequency data, and thus the p‐distance calculations were conducted with a simplified data set that included exactly one copy of each allele, that is, assuming equal allele frequencies. In actuality, the allele frequencies are unlikely to all be equal. The direction and magnitude in which this would impact the estimate of p‐distance would depend on the relative divergence of the high‐ and low‐frequency alleles, which of course is unknown in the absence of allele frequency data.

We measured variability in deduced amino acid sequences using the same approach. After translating the trimmed nucleotide sequences and removing any nonunique amino acid alleles, we counted the number of unique alleles and computed average p‐distance in MEGA X using pairwise deletion. As with the above approach for nucleotide data, this makes the necessary simplifying assumption that the alleles occur at equal frequencies.

We then explored whether the number of MHC‐IIA alleles might vary across species in relation to the scope of sampling and/or aspects of evolutionary history. Specifically, we ran a generalized linear model (GLM) to test for main effects of the following fixed‐effects predictor variables: sample size (NumAnimals; continuous), number of genes assayed (NumGenes; continuous; as determined by the original authors of each data set), and taxonomy (Taxon; categorical: fish, amphibian, bird, mammal). The number of unique nucleotide sequences (NumAllelesDNA) was modeled as a negative binomial GLM with a log link:

$${\text{NumAllelesDNA}}_{i}\;∼\;\text{NegBin}\;\left(\mu_{i},k\right)$$

$$E\left({\text{NumAllelesDNA}}_{i}\right)=\mu_{i}\;\text{and}\;\mathrm{var}\left({\text{NumAllelesDNA}}_{i}\right)=\mu_{i}+\left({\mu_{i}}^{2}/k\right)$$

$$\log\;\left(\mu_{i}\right)=\eta_{i}$$

$$\eta_{i}=\beta_{1}+\beta_{2}\;x\;{\text{NumAnimals}}_{i}+\beta_{3}\;x\;{\text{NumGenes}}_{i}+\beta_{4}\;x\;{\text{Taxon}}_{i}$$

### 2.5 Positive selection on MHC‐IIA

Within species, we tested whether MHC‐IIA exon 2 sequences showed signatures of positive selection. We used EasyCodeML (Gao et al., 2019) to implement site models in the CodeML program in PAML (Yang, 2007), comparing models that include different amounts of purifying selection, neutral evolution, and positive selection.

First, we built a phylogenetic tree for the trimmed, aligned MHC‐IIA nucleotide sequences for each species separately. As described above, to accommodate the large number of species in which orthology or gene identity of alleles was unknown, we combined all MHC‐IIA alleles from a given species into a single alignment. We used MEGA X to build a maximum likelihood tree, using a general time reversible model, with gamma‐distributed rates among sites plus invariant sites, and pairwise deletion (MEGA's “use all sites”) to accommodate indels. This tree and the alignment were then used as inputs for EasyCodeML.

We used the Preset Mode in EasyCodeML to run different site models, comparing the null model M7 against the alternate model M8. Null model M7 is a nearly neutral model in which the values of ω (the ratio of nonsynonymous to synonymous substitutions, dN/dS) follow an empirically determined beta distribution bounded by 0 and 1 that describes a mix of neutral evolution and purifying selection across sites. In the alternate model M8, additional parameters allow some codons to evolve by positive selection. Specifically, a beta distribution is fit to a subset of codons that are empirically determined to be evolving via neutral evolution or purifying selection, while a different subset of codons is empirically identified as undergoing positive selection in which ω_s_ is estimated from the data as some value >1 (Yang et al., 2005).

We extracted three key pieces of output data:

- the overall evidence that a model with positive selection is a better fit than a nearly neutral model, i.e. the statistic of the likelihood‐ratio test: 2(log[L(M8)] – log[L(M7)])
- under M8, the proportion of codons that are estimated to be under positive selection (i.e. p_1_ of M8)
- under M8, the magnitude of dN/dS at those codons that are estimated to be under positive selection (i.e. ω_s_ of M8)

We then explored whether the support for M8 might vary across species in relation to the scope of sampling and/or aspects of evolutionary history. Specifically, we ran a generalized linear model to test for main effects of the following fixed‐effects predictor variables: sample size (NumAnimals; continuous), number of genes assayed (NumGenes; continuous), and taxonomy (Taxon; categorical: fish, amphibian, bird, mammal). The statistic of the likelihood ratio test of CodeML's model M8 as an alternative to the null hypothesis M7 (M8_LnL) was modeled as a gamma GLM with a log link:

$$M8\_{\mathrm{LnL}}_{i}\;∼\;\text{Gamma}\;\left(\mu_{i},\tau \right)$$

$$E\left(M8\_{\mathrm{LnL}}_{i}\right)=\mu_{i}\;\text{and}\;\mathrm{var}\left(M8\_{\mathrm{LnL}}_{i}\right)=\left(\mu_{i}/\tau \right)$$

$$\log\;\left(\mu_{i}\right)=\eta_{i}$$

$$\eta_{i}=\beta_{1}+\beta_{2}\;x\;{\text{NumAnimals}}_{i}+\beta_{3}\;x\;{\text{NumGenes}}_{i}+\beta_{4}\;x\;{\text{Taxon}}_{i}$$

### 2.6 MHC‐IIA diversity and selection: Comparisons with MHC‐IIB

For a subset of the species with MHC‐IIA data, we were able to find MHC‐IIB data from the same individuals or populations. This allowed a test of whether MHC‐IIA and MHC‐IIB differ in number of alleles or in evidence for positive selection, when controlling for the other possible sources of interspecific variation described above. Thus, for these two response variables (NumAllelesDNA and M8_LnL) we compared MHC‐IIA and MHC‐IIB by running generalized linear mixed models (GLMMs). These models were similar to the GLMs outlined above except that we included two additional predictor variables: Class as a fixed effect (categorical: IIA, IIB) and Species as a random intercept to account for dependency of MHC‐IIA and MHC‐IIB data from the same species. This GLMM structure allowed us to ask if a species' MHC‐IIA and MHC‐IIB differ in diversity or selection history, when controlling for potential effects of sample size (NumAnimals), number of genes amplified (NumGenes), and taxonomic group (Taxon: mammal, bird, amphibian, fish).

The number of unique nucleotide sequences (NumAllelesDNA) was modeled as a Poisson GLMM with a log link:

$${\text{NumAllelesDNA}}_{\mathrm{ij}}\;∼\;\text{Poisson}\;\left(\mu_{\mathrm{ij}}\right)$$

$$E\left({\text{NumAllelesDNA}}_{\mathrm{ij}}\right)=\mu_{\mathrm{ij}}\;\text{and}\;\mathrm{var}\left({\text{NumAllelesDNA}}_{\mathrm{ij}}\right)=\mu_{\mathrm{ij}}$$

$$\log\;\left(\mu_{\mathrm{ij}}\right)=\eta_{\mathrm{ij}}$$

$$\begin{array}{cc} \eta_{\mathrm{ij}}= & \beta_{1}+\beta_{2}\;x\;{\text{NumAnimals}}_{\mathrm{ij}}+\beta_{3}\;x\;{\text{NumGenes}}_{\mathrm{ij}}\\ & +\beta_{4}\;x\;{\text{Taxon}}_{\mathrm{ij}}+\beta_{5}\;x\;{\text{Class}}_{\mathrm{ij}}+{\text{Species}}_{j}+\varepsilon_{\mathrm{ij}} \end{array}$$

$${\text{Species}}_{j}\;∼\;N\left(0,{\sigma^{2}}_{\text{Species}}\right)$$

$$\varepsilon_{\mathrm{ij}}\;∼\;N\left(0,\sigma^{2}\right)$$

Evidence for positive selection – i.e. the statistic of the likelihood ratio test of CodeML's model M8 as an alternative to the null hypothesis M7 (M8_LnL) – was modeled as a gamma GLMM with a log link:

$$M8\_{\mathrm{LnL}}_{i}\;∼\;\text{Gamma}\;\left(\mu_{i},\tau \right)$$

$$E\left(M8\_{\mathrm{LnL}}_{i}\right)=\mu_{i}\;\text{and}\;\mathrm{var}\left(M8\_{\mathrm{LnL}}_{i}\right)=\left(\mu_{i}/\tau \right)$$

$$\log\;\left(\mu_{i}\right)=\eta_{i}$$

$$\begin{array}{cc} \eta_{\mathrm{ij}}= & \beta_{1}+\beta_{2}\;x\;{\text{NumAnimals}}_{\mathrm{ij}}+\beta_{3}\;x\;{\text{NumGenes}}_{\mathrm{ij}}\\ & +\beta_{4}\;x\;{\text{Taxon}}_{\mathrm{ij}}+\beta_{5}\;x\;{\text{Class}}_{\mathrm{ij}}+{\text{Species}}_{j}+\varepsilon_{\mathrm{ij}} \end{array}$$

$${\text{Species}}_{j}\;∼\;N\left(0,{\sigma^{2}}_{\text{Species}}\right)$$

$$\varepsilon_{\mathrm{ij}}\;∼\;N\left(0,\sigma^{2}\right)$$

GLMs and GLMMs were run in R 4.1.1 (R Core Team, 2021) with the lme4 (Bates et al., 2015), MASS (Venables & Ripley, 2002), contrast (Kuhn et al., 2021), multcomp (Hothorn et al., 2008), and emmeans (Lenth, 2022) packages. Code is available on Dryad as described in the Data Availability statement. Significance of predictor variables was assessed by single‐term deletion, dropping them individually from the model and testing for worse fit using a likelihood test. Differences between levels of significant categorical variables (i.e., taxon) were assessed with post hoc contrasts with unadjusted *p*‐values.

GLMs and GLMMs were fitted and validated by assessing the following: over‐ or under‐dispersion; excess zeroes in the response variable; multicollinearity among the predictors; departures from linearity in Q‐Q plots; patterns in plots of residuals versus fitted values, scale versus location, and residuals versus leverage; values of Cook's Distance; and patterns in plots of residuals versus predictors.

## 3 RESULTS

### 3.1 Prevalence of MHC‐IIA versus MHC‐IIB in studies of sexual selection or infectious disease

**FIGURE 2:**
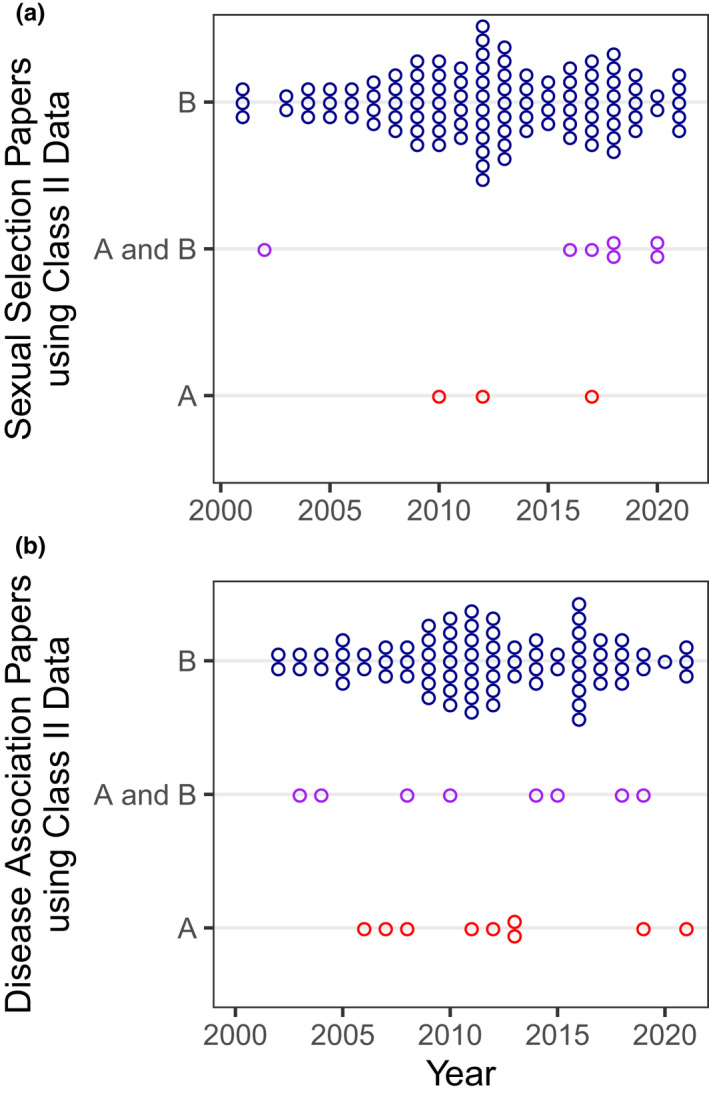
Prevailing use of MHC‐IIB and MHC‐IIA data in empirical studies of sexual selection (top) and disease (bottom) published between 2000 and 2021. Each circle represents one study. In both sets of papers, the prevalence of MHC‐IIA data has not increased significantly over the past two decades (see text). (a) Studies of sexual selection, classified by whether a paper's data includes MHC‐IIA (*n* = 3), IIA and IIB (*n* = 7), or just MHC‐IIB (*n* = 106). In total, 97% of the 116 class II sexual selection papers examined MHC‐IIB data, whereas only 9% examined MHC‐IIA data. (b) Studies of disease associations, classified by whether a paper's data includes MHC‐IIA (*n* = 9), IIA and IIB (*n* = 8), or just MHC‐IIB (*n* = 78). In total, 91% of the 95 class II disease papers examined MHC‐IIB data, whereas only 18% examined MHC‐IIA data.

Our literature search found that ecological studies of class II MHC have indeed heavily focused on MHC‐IIB at the expense of MHC‐IIA (Figure 2). In studies of sexual selection and class II MHC, the overwhelming majority of papers included only MHC‐IIB data (*n* = 106), versus studies that examined MHC‐IIA (*n* = 3) or both IIA and IIB (*n* = 7). Put another way, 97% of the 116 class II sexual selection papers examined MHC‐IIB data, whereas only 9% examined MHC‐IIA data. The use of MHC‐IIA data in class II sexual selection studies has not increased with time (no significant effect of publication year in logistic regression: likelihood X^2^_1_ = 2.64, *p* = .104, pseudo‐R^2^ = 0.04; Table S1).

Likewise, studies of disease associations have disproportionately focused solely on MHC‐IIB (*n* = 78 papers), versus those that examined MHC‐IIA (*n* = 9) or both IIA and IIB (*n* = 8). In total, 91% of the 95 class II disease papers examined MHC‐IIB data, whereas only 18% examined MHC‐IIA data. The use of MHC‐IIA data in class II disease studies has not increased with time (no significant effect of publication year in logistic regression: likelihood X^2^_1_ < 0.01, *p* = .99, pseudo‐R^2^ < 0.01; Table S2).

### 3.2 MHC‐IIA diversity and selection: Summary of MHC‐IIA data sets

We found MHC‐IIA studies of 50 vertebrate species that met our inclusion criteria for analysis of diversity (see Appendix 1 and Methods; e.g., including studies which found just one allele in a species). The median sample size of analysed individuals was 26 (range: 2–674), and the number of genes assayed ranged from 1 to 4. Mammals were the most commonly studied group (*n* = 31), followed by bony fish (*n* = 13), birds (*n* = 4), and amphibians (*n* = 2). Of those 50 data sets, 42 contained at least three alleles (the required minimum number of alleles for CodeML) and thus were also amenable to CodeML tests of positive selection. We refer readers to Section 2 of the Supporting Information for detailed comments on our decision to pool alleles across genes within species.

### 3.3 Allelic diversity in MHC‐IIA

**FIGURE 3:**
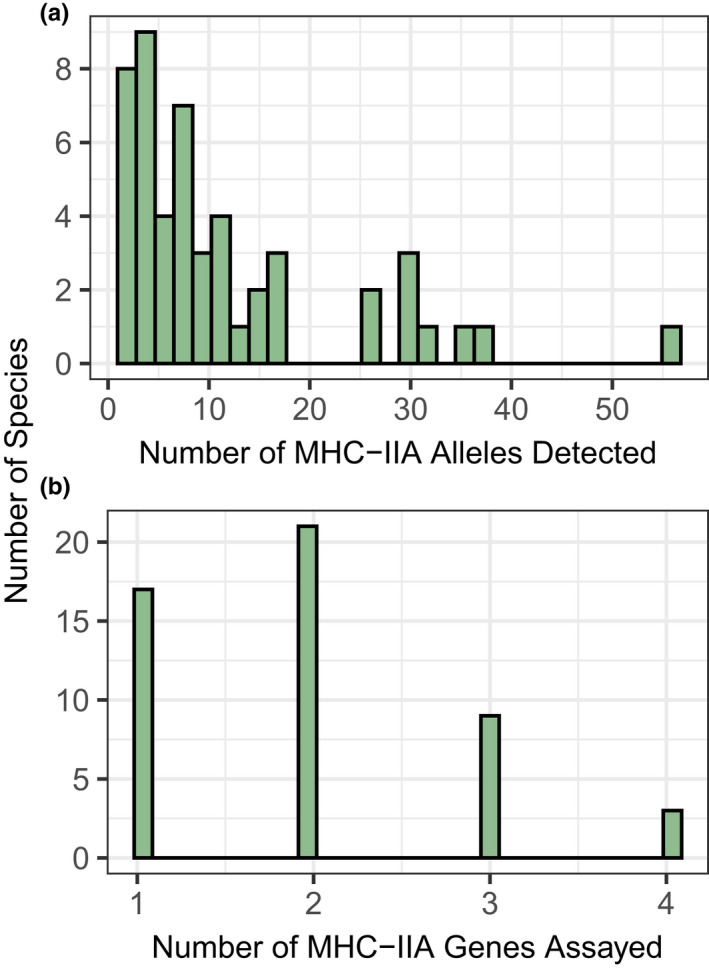
MHC‐IIA variability across the 50 analysed data sets. (a) At the allelic level, the detected number of unique MHC‐IIA exon 2 nucleotide sequences ranged from 1 to 55 across 50 species, with a median of 8. (b) At the gene level, the data sets from the top panel each assayed between 1 and 4 MHC‐IIA genes.

**FIGURE 4:**
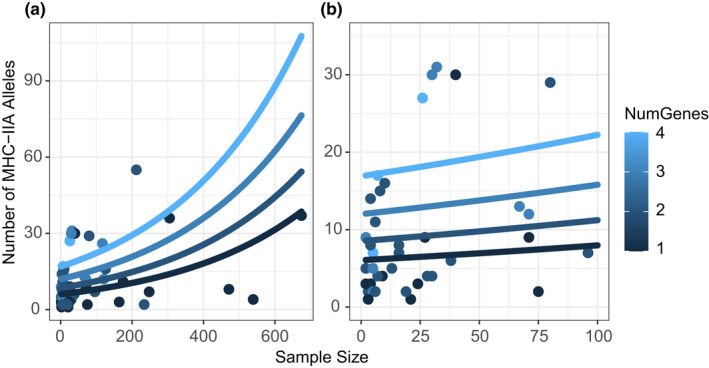
Relationship between sample size and observed MHC‐IIA variability in published vertebrate data sets. Observed values (dots) and GLM fitted values (lines) for number of IIA alleles in a species (i.e., number of unique exon 2 nucleotide sequences). (a) Full range of data, *n* = 50 species. (b) Magnified view of both axes for better readability of the 38 studies with sample sizes <100. In the GLM, number of alleles covaried positively with sample size and with number of genes assayed (NumGenes) but did not differ among taxonomic groups (Taxon: mammal, bird, amphibian, fish. The fitted values shown by the lines are averaged across levels of Taxon, weighted by the relative frequency of mammals, birds, amphibians, and fish in the raw data. Sample size (x‐axis) and number of genes (colour coding) vary across the ranges observed in the raw data. See Table S3 for GLM parameters.

The number of MHC‐IIA alleles varied widely across data sets, with a median of two genes assayed and eight unique exon 2 nucleotide sequences detected per species (quartiles: 4, 15; Figure 3). We used a GLM to test three variables that might explain some of this interspecific variation in number of alleles. The number of MHC‐IIA alleles reported in a species covaried positively with sample size (GLM likelihood ratio X^2^_df = 1_ = 11.1, *p* = .001) and with number of genes assayed (GLM likelihood ratio X^2^_df = 1_ = 6.3, *p* = .012), but there was no significant variation among taxonomic groups (mammal, bird, amphibian, fish; GLM likelihood ratio X^2^_df = 3_ = 6.5, *p* = .088). Explained deviance for the model was 30.8% (Figure 4, Table S3). A separate GLM of only mammals found significant differences in MHC‐IIA diversity between the six mammalian orders represented in our data sets (Figure S1, Table S4).

Within species, the reported MHC‐IIA alleles were diverse: the mean p‐distance between a species' alleles was 0.125 ± 0.101 SD for nucleotide sequences, and 0.191 ± 0.130 SD for inferred amino acid sequences (Figure S2). We also found that the number of amino acid sequences within a species almost perfectly mirrored the underlying number of DNA haplotypes, with a slope of 0.959 (95% CI: 0.926–0.991) and an r^2^ of 0.986 (Figure S3).

### 3.4 Positive selection on MHC‐IIA

MHC‐IIA sequences showed evidence of positive selection in most species when assessed with CodeML. We measured the statistic of the likelihood ratio test of model M8, which incorporates positive selection at an empirically determined subset of codons, versus null model M7, which allows only purifying selection and drift. In 30 of 42 vertebrate species (71%), the evidence for M8 exceeded the critical value of X^2^_df = 2_ = 5.99 (Figure S4). Across the 42 species, the proportion of MHC‐IIA exon 2 codons estimated as being under positive selection (i.e., p_1_ of M8) had a median value of 0.188 (Figure S4). For those codons classified by M8 as being under positive selection, the median value for ω_s_ of M8 was 5.4 (Figure S4).

**FIGURE 5:**
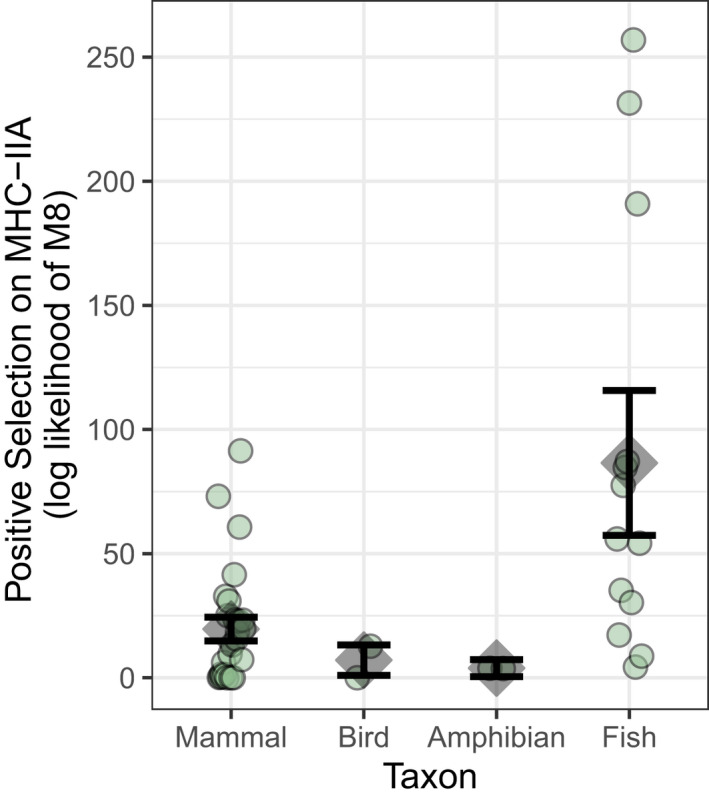
Signals of positive selection at MHC‐IIA in vertebrates. Observed values (green dots, *n* = 42) and estimated marginal means (grey diamonds ± SE) from a GLM analysing the statistic of the likelihood ratio test of CodeML's model M8 as an alternative to the null hypothesis M7 for IIA in 42 species. This evidence of positive selection on IIA was greater in fish than in other taxonomic groups, but was not significantly related to sample size or number of genes assayed. Points are horizontally dodged for clarity. Estimated marginal means by taxonomic group account for effects of other covariates in the model. See Table S5 for GLM parameters.

We used a GLM to test three variables that might explain some of the interspecific variation in the evidence for positive selection on MHC‐IIA. The support for CodeML's model M8 differed between taxonomic groups (GLM likelihood ratio X^2^_df = 3_ = 19.9, *p* = .0002) but did not covary with sample size (GLM likelihood ratio X^2^_df = 1_ = 1.7, *p* = .190) or number of genes assayed (GLM likelihood ratio X^2^_df = 1_ = 0.3*, p* = .584). Explained deviance for the model was 27.5% (Table S5). Post‐hoc contrasts across the four taxonomic groups found stronger evidence for positive selection in fish than in any other group (*p* = .001 vs. mammals, *p* = .010 vs. birds, *p* = .002 vs. amphibians), while mammals, amphibians, and birds did not differ from each other (all *p* > .08; Figure 5).

### 3.5 MHC‐IIA diversity and selection: Comparisons with MHC‐IIB

Of the 50 species with MHC‐IIA data, 27 also had MHC‐IIB data from the same individuals or populations. Of those joint IIA / IIB data sets, 20 of 27 species had enough alleles at both IIA and IIB for CodeML analysis of both types of genes. Although we restricted our MHC‐IIB data sets to those drawn from at least the same population as the MHC‐IIA data, the corresponding studies frequently differed in sample size (7 of 27 studies) or in number of genes assayed (12 of 27 studies). Thus, to test robustly for differences in polymorphism or selection in MHC‐IIA versus MHC‐IIB, we fitted GLMMs to compare IIA and IIB while accounting for potential effects of those other variables.

**FIGURE 6:**
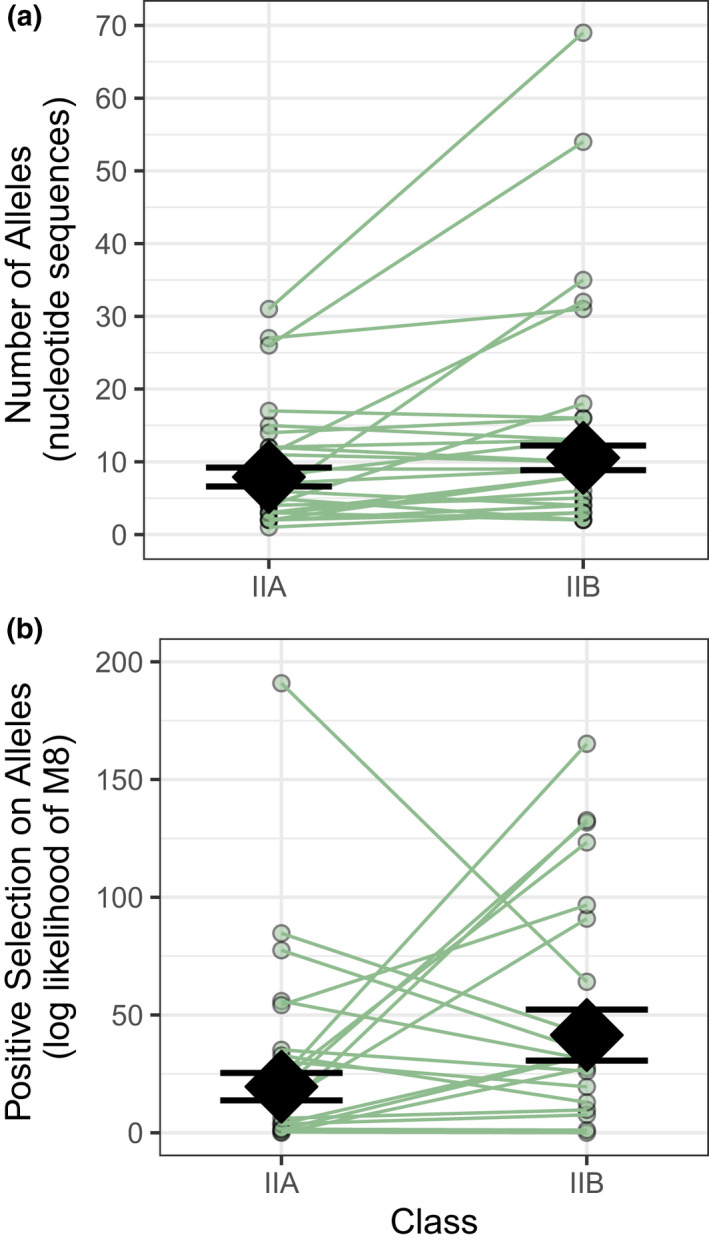
Comparison of MHC‐IIA and MHC‐IIB diversity (a) and positive selection (b). Observed values (small green dots, with lines connecting data from same species) and estimated marginal means (large black diamonds, ± SE) from GLMMs are shown, to account for effects of other covariates. (a) Number of unique class II exon 2 nucleotide sequences in a species. See Table S6 for GLMM parameters. (b) Evidence for positive selection in a species, as measured by the statistic of the likelihood ratio test of CodeML's model M8 as an alternative to the null hypothesis M7. See Table S7 for GLMM parameters. In both GLMMs, the response variable was modeled as a function of fixed main effects of Class (IIA, IIB), sample size, number of genes assayed, and taxonomic group (mammal, bird, amphibian, fish). Species was used as a random effect to account for dependencies in IIA and IIB data sets from the same species.

Although MHC‐IIA was polymorphic, it was roughly 25% less so than MHC‐IIB, as assessed in a Poisson GLMM with log link. Among 27 species with appropriate IIA and IIB polymorphism data, the number of unique exon 2 nucleotide sequences was lower in MHC‐IIA than in MHC‐IIB (GLMM likelihood ratio X^2^_df = 1_ = 10.9, *p* = .0009), when accounting for positive effects of sample size (GLMM likelihood ratio X^2^_df = 1_ = 24.1, *p* = .0001), number of genes assayed (GLMM likelihood ratio X^2^_df = 1_ = 20.1, *p* = .0001), and differences among taxonomic groups (GLMM likelihood ratio X^2^_df = 3_ = 10.0, *p* = .0183) (Figure 6, Figure S5, Table S6). The overall model had a pseudo‐R^2^ of 0.52 for fixed effects (NumAnimals, NumGenes, and Taxon), and a total pseudo‐R^2^ of 0.86 when including the random effect of species. More concretely, a species had an average of roughly three fewer alleles in MHC‐IIA than in MHC‐IIB (marginal means ± SE: 7.9 ± 1.3 for IIA, vs. 10.6 ± 1.7 for IIB). Post‐hoc contrasts on taxonomic groups also found that fish and amphibians both had roughly twice as many class II alleles as mammals (z = 3.10, *p* = .002 and z = 1.98, *p* = .048, respectively; Figure S5), while no other pairwise comparisons between taxonomic groups were significant (all *p* > .16).

Using a similar mixed‐model approach, we found that signals of positive selection were stronger for MHC‐IIB than for MHC‐IIA. With 40 matched data sets from 20 species, we fit a GLMM to predict variation between data sets in the statistic of the likelihood ratio test of CodeML's model M8 as an alternative to the null hypothesis M7. This evidence for positive selection was roughly twice as strong for MHC‐IIB than for MHC‐IIA (GLMM likelihood ratio X^2^_df = 1_ = 5.0, *p* = .0254) when controlling for positive effects of sample size (GLMM likelihood ratio X^2^_df = 1_ = 7.6, *p* = .0057), number of genes assayed (GLMM likelihood ratio X^2^_df = 1_ = 12.8, *p* = .0004), and differences among taxonomic groups (GLMM likelihood ratio X^2^_df = 3_ = 20.7, *p* = .0001) (Figure 6, Figure S6, and Table S7). Marginal and conditional pseudo‐R^2^ for the model were both 0.63, because the random effect of species did not account for a measurable amount of variation in the response variable (unlike the GLMM of number of alleles, where species was important). Post‐hoc contrasts on taxonomic groups found stronger evidence for positive selection in fish than in mammals (*p* < .0001) or amphibians (*p* = .0004); other pairwise contrasts were not significant (all *p* > .05; Figure S6).

## 4 DISCUSSION

As a starting point, our literature summary provided a quantitative confirmation that the genes encoding the alpha subunit of class II MHC have received very little attention, compared to MHC‐IIB, in studies of functional variation such as sexual selection or disease associations (Figure 2). Furthermore, this attention to MHC‐IIA has not increased markedly over the past two decades. This observed scarcity of MHC‐IIA data in studies of sexual selection or disease cannot be entirely attributed to a lack of PCR primers or other foundational studies on IIA: a search for basic characterizations of MHC‐IIA genes found studies of 50 different vertebrate species that met our inclusion criteria for assessing polymorphism and positive selection. Our analyses of those 50 species found that MHC‐IIA is often polymorphic and shows signatures of positive selection, making the case that studies of ecology, evolution, and conservation in MHC class II should incorporate IIA much more often.

### 4.1 Conclusions on MHC‐IIA variability and patterns of selection

The first broad conclusion from our analysis of those DNA sequence data sets is that MHC‐IIA is often polymorphic, with a median of eight and as many as 55 alleles reported. Not surprisingly, the extent of polymorphism varied widely across studies. This interspecific variation in the number of MHC‐IIA alleles was not driven by any consistent differences between mammals, birds, amphibians, and fish (although there was variation between mammalian orders – see Figure S1, Table S4). However, the number of MHC‐IIA alleles covaried positively with the number of animals sampled, and also with the number of genes assayed. The most likely explanation for the relationship with sample size is simply that greater sampling effort yields more alleles, akin to a species accumulation curve in ecology (Gotelli & Colwell, 2001). In contrast to this straightforward sample size effect, the relationship between number of alleles found and number of genes assayed is more challenging to interpret, as researchers often do not know whether the number of MHC‐IIA genes being assayed is representative of the number of MHC‐IIA genes actually present in the genome. One possible interpretation of the positive relationship between number of alleles found and number of genes assayed is a sampling artefact: studies find more alleles if they assay a greater proportion of the (known or unknown number of) existing genes. This explanation is valid only if the number of MHC‐IIA genes assayed in a given study does not tightly correspond to the true number of IIA genes in the focal species' genome. An alternative explanation is that the true number of IIA alleles and the true number of IIA genes in a species might covary biologically. For example, evolutionary forces favouring MHC diversification in a species or lineage could lead to gene duplication and subsequent divergence, producing both more genes and more unique alleles over time.

The second main conclusion is that MHC‐IIA genes within a species usually showed strong signatures of positive selection, rather than evolving solely via purifying selection and drift. Our CodeML analyses found that, in over 70% of species, MHC‐IIA alleles exhibited a significantly better fit to an evolutionary model with positively selected sites than to a null model that allows only purifying selection and drift. Furthermore, a median of 19% of sites in exon 2 were identified as being under positive selection, and the estimated value of dN/dS at these positively selected sites was high, with a median value of 5.4.

As with allelic polymorphism, there was substantial variation among species in the strength of support for positive selection on MHC‐IIA. However, the predictors of that interspecific variation were not the same as for IIA polymorphism levels. The signal strength of positive selection on MHC‐IIA in a species showed no effect of sample size or number of genes assayed, but was stronger in fish than in mammals, birds, or amphibians. The lack of effect of sample size and number of genes might mean that even a relatively small sample of a species' alleles captures enough of the phylogenetic diversity of MHC sequences to yield a meaningful test for positive selection.

The detection of stronger evidence of positive selection on MHC‐IIA in fish than in other groups has several candidate interpretations. One centres on aquaculture. Eight of the 13 IIA data sets from fish were from hatchery or aquaculture settings, where the high density of a single fish species and the spatial overlap of adults and juveniles can set the stage for more pronounced disease transmission (Krkošek et al., 2006; Pulkkinen et al., 2010). If enough time elapses, the resulting pathogen‐mediated selection on MHC (Cao et al., 2018; Yang et al., 2016) might not only alter allele frequencies of standing genetic variation, but also lead to signals of positive selection. There might also be direct artificial selection for parasite‐resistant traits in aquaculture species (Kube et al., 2012), given that the proportion of farmed or domesticated species in our data set was somewhat higher in fish (8 of 13 species) than in mammals (9 of 31 species). Another explanation for greater positive selection in fish could be the different genome structure of teleosts, in which the major histocompatibility genes are not packed into a dense complex (Grimholt, 2016; Stet et al., 2003). In particular, the lack of linkage between class I and class II genes in fish can decouple how those genes evolve, which might create more scope for MHC‐IIA to respond to pathogen‐mediated selection even in the presence of strong selection on class I from different pathogens.

The third main conclusion from our analyses is that although MHC‐IIA generally exhibits polymorphism and a history of positive selection, both phenomena are more pronounced in the population‐matched IIB data sets that we examined. In the subset of 27 species with appropriate data, studies of MHC‐IIB reported an average of three more alleles than studies of MHC‐IIA (10.6 vs. 7.9) when controlling for potential effects of sample size, number of genes assayed, and taxonomic group. Likewise, in the subset of 20 species amenable to CodeML analysis for both types of genes, MHC‐IIB showed stronger evidence for positive selection than MHC‐IIA when controlling for those same covariates. Given the tight interaction of alpha and beta subunits in the MHC heterodimer (Figure 1), and the key role of the MHC in immune defence and mate choice, our findings nevertheless underscore the importance of examining both IIB and IIA sequences in such studies. As genomic organization of the MHC becomes better understood in a wider range of species, it should become possible to test whether polymorphism and diversifying selection in MHC‐IIA are more evident in A‐B gene pairs that experience low recombination rates, as predicted by the idea that high A‐B recombination constrains one subunit to a “best average fit” with the alleles of the other, more polymorphic, subunit (Kaufman, 1999).

### 4.2 Calls for future studies

Class IIA of the MHC clearly warrants more attention. Our analysis of MHC‐IIA from 50 species of vertebrates provides variable but important evidence for (i) allelic polymorphism and (ii) a history of positive selection. This evidence is in line with theoretical expectations, given the contribution of IIA to the peptide binding groove, but is strongly at odds with the widespread lack of IIA data in studies of the functional importance of class II diversity. This leads us to make three broad calls for future studies.

First, we encourage researchers to characterize allelic polymorphism and selection history in MHC‐IIA in additional species. Given that 44 of the 50 MHC‐IIA data sets in our meta‐analysis came from mammals and bony fish, future studies should give increased attention to other taxa, for example, birds, other reptiles, amphibians, and cartilaginous fish.

Second, we need to better ascertain the number, location, and orthology of MHC‐IIA genes as part of studying MHC genomics in a wide range of taxa. The known variation in MHC genomics – even within major vertebrate groups, such as between songbirds and chicken *Gallus domesticus* (O'Connor et al., 2019) or between Atlantic salmon *Salmo salar* and Atlantic cod *Gadus morhua* (Grimholt, 2016) – emphasizes the shortcomings in what is known about most other species. As can be true of MHC‐IIB, work on the evolution of MHC‐IIA alleles is hampered by this lack of fundamental genomic knowledge. For example, if a study cannot assign MHC‐IIA alleles to specific MHC‐IIA genes, then it becomes impossible to answer questions about allele frequencies and mechanisms of balancing selection (Phillips et al., 2018) or about lineages of alleles over deep time (Goebel et al., 2017). Although such work has progressed much further in mammals, where characterization and annotation of MHC‐IIA lineages is far advanced (e.g., Amills et al., 2008), we found only four mammalian species with sufficient data for measuring MHC‐IIA polymorphism in all three genes (DPA, DQA, DRA), and only two of those species had complete data for assessing positive selection. Our analysis therefore could not break down MHC‐IIA variability to gene/lineage level, not even in mammals. More broadly, additional comparative data on MHC genomics are required to understand not only orthology but also whether MHC‐IIA resembles MHC‐IIB in phenomena such as gene expansion (He et al., 2021) and copy number variation (Minias et al., 2019; Talarico et al., 2022).

To tackle these genomic questions, MHC research should take advantage of improvements in long‐read sequencing technologies, as several recent studies have done (Cheng et al., 2021; Fuselli et al., 2018; He et al., 2021). PacBio and NanoPore sequencing now can generate reads from 10s to 100s of kb (Amarasinghe et al., 2020; Hon et al., 2020), circumventing the problems inherent in making MHC assemblies from short sequencing reads (He et al., 2021). A wider understanding of MHC gene identity, lineage history, and genomic organization would greatly enhance our ability to explore patterns of polymorphism and selection in MHC‐IIA, and should lead to a clearer picture of important phenomena such as linkage, gene duplication, and copy number variation.

Third, an important area for future studies is to expand our understanding of class II heterodimer formation. Molecular characterization of MHC‐IIA genes is an important step, but ultimately a functional perspective on class II MHC will require an improved understanding of the joint action of the alpha and beta subunits of the protein. This will be most easily achievable in species with only a single classical MHC‐IIA and MHC‐IIB gene (e.g., Atlantic salmon) (Grimholt, 2016), but will be more complicated in other taxa. In species with a single MHC‐IIA and multiple MHC‐IIB genes (e.g., chicken), the alpha subunit may be evolutionarily constrained by the need to work effectively with different beta subunits – that is, a best average fit (Salomonsen et al., 2003). In other species, there are multiple MHC‐IIA and multiple MHC‐IIB genes (e.g., Leach's storm‐petrel, *Hydrobates leucorhous*), raising the question of whether specific A‐B gene combinations always pair in a heterodimer and thus might coevolve as a team (Rand et al., 2019). Outside of a few primate and rodent models (Askew & Harding, 2002; Braunstein & Germain, 1987; Temme et al., 2019), we currently lack data to investigate these questions.

### 4.3 Outlook

Our meta‐analysis shows that – despite their historical underrepresentation in immunogenetic studies – MHC‐IIA genes are nearly as polymorphic as MHC‐IIB and show clear signals of positive selection. Undoubtedly, there are some species where MHC‐IIA diversity is quite limited and where a strong focus on MHC‐IIB is probably justified, but researchers can no longer simply assume that this is the case in less well‐studied species. We therefore suggest that it is conceptually important to incorporate MHC‐IIA data into studies of pathogen‐mediated selection, mate choice, and other aspects of ecology and evolution. The class II peptide binding groove is encoded by two types of genes, and it is time to consider them both to gain an integrative understanding of their functional importance.

## AUTHOR CONTRIBUTIONS

Donald C. Dearborn designed research, performed research, analysed data, and wrote the manuscript. Sophie Warren performed research and contributed to writing the manuscript. Frank Hailer designed research and wrote the manuscript.

## CONFLICT OF INTEREST

The authors declare no conflict of interest.

## BENEFIT‐SHARING STATEMENT

Benefits generated: Benefits from this research accrue from the sharing of our data and R scripts on public databases as described above.

## Supporting information


**Appendix S1** Supporting information

## Data Availability

The DNA sequence data were retrieved from published studies as listed in Appendix 1. Our single‐species alignments, measures of polymorphism and positive selection, associated covariates, and R scripts for the GLMs and GLMMs are available on DataDryad [data set] (Dearborn et al., 2022) at https://doi.org/10.5061/dryad.fbg79cnx0.

## APPENDIX 1 Key variables for 50 species with MHC‐IIA data that met our inclusion criteria. See Data Availability statement for .csv file of complete MHC‐IIA and MHC‐IIB data set

**Table:**

Class and order	Family	Common Name	Latin Name	MHC‐IIA No. of Animals^a^	MHC‐IIA No. of Genes^b^	MHC‐IIA No. of Alleles^c^	IIA MHC‐IIA M8 Log Likelihood^d^	MHC‐IIA Source	MHC‐IIB Source, if any
Mammalia: Primates	Cercopithecidae	Rhesus Macaque	*Macaca mulatta*	30	3	30	23.2	Deng et al. (2013)	N/A
		Cynomolgus Macaque	*Macaca fascicularis*	117	3	26	18.5	Blancher et al. (2014)	Blancher et al. (2012, 2014)
		Pigtailed Macaque	*Macaca nemestrina*	32	3	31	23.3	Karl et al. (2014)	Karl et al. (2014)
	Hominidae	Chimpanzee	*Pan troglodytes*	67	3	13	15.6	Otting et al. (2019)	N/A
	Cheirogaleidae	Grey Mouse Lemur	*Microcebus murinus*	10	2	16	41.5	Averdam et al. (2011)	N/A
Mammalia: Lagomorpha	Leporidae	European Rabbit	*Oryctolagus cuniculus*	40	1	30	91.4	Magalhães et al. (2015)	N/A
		European Brown Hare	*Lepus europaeus*	674	1	37	60.7	Koutsogiannouli et al. (2009)	N/A
Mammalia: Rodentia	Cricetidae	Common Vole	*Microtus arvalis*	16	2	8	30.9	Bryja et al. (2006)	N/A
		Bank Vole	*Clethrionomys glareolus*	16	2	7	25	Bryja et al. (2006)	N/A
		Water Vole	*Arvicola terrestris*	96	2	7	9.5	Bryja et al. (2006)	N/A
	Muridae	Australian Bush Rat	*Rattus fuscipes greyii*	305	1	36	73.1	Seddon and Baverstock (1999)	N/A
Mammalia: Artiodactyla	Bovidae	Bighorn Sheep	*Ovis canadensis*	24	1	3	0	Subramaniam et al. (2012)	Subramaniam et al. (2012)
		Domestic Sheep	*Ovis aries*	126	2	16	13.2	Snibson et al. (1998)	N/A
		Gayal	*Bos frontalis*	3	2	2		Memon et al. (2018)	N/A
		Water Buffalo	*Bubalus bubalis*	75	1	2		Sena et al. (2003)	Sena et al. (2003)
		Yak	*Bos grunniens*	6	2	2		Ge et al. (2016)	N/A
	Moschidae	Forest Musk Deer	*Moschus berezovskii*	71	3	12	1.3	Yao et al. (2015)	Yao et al. (2015)
	Suidae	Domestic Pig	*Sus scrofa*	471	1	8	7.3	Liu et al. (2015)	N/A
	Camelidae	Wild Camel	*Camelus ferus*	13	2	5	0.4	Plasil et al. (2016)	Plasil et al. (2016)
		Bactrian Camel	*Camelus bactrianus*	38	2	6	0.3	Plasil et al. (2016)	Plasil et al. (2016)
		Dromedary	*Camelus dromedarius*	28	2	4	1.5	Plasil et al. (2016)	Plasil et al. (2016)
Mammalia: Perissodactyla	Equidae	Plains Zebra	*Equus quagga*	71	1	9	0	Kamath and Getz (2011)	N/A
		Balkan Donkey	*Equus asinus africanus*	248	1	7	0.1	Arbanasic̈ et al. (2013)	N/A
		Domestic Horse	*Equus caballus*	539	1	4	0	Brown et al. (2004)	N/A
Mammalia: Carnivora	Mustelidae	European Badger	*Meles meles*	7	2	4	6.1	Sin et al. (2012)	Sin et al. (2012)
		Sea Otter	*Enhydra lutris*	3	1	1		Bowen et al. (2006)	Bowen et al. (2006)
	Otariidae	California Sea Lion	*Zalophus californianus*	19	2	2		Bowen et al. (2002)	Bowen et al. (2002)
	Ursidae	Giant Panda	*Ailuropoda melanoleuca*	121	3	12	32.8	Chen et al. (2010)	Chen et al. (2010)
	Canidae	African Wild Dog	*Lycaon pictus*	234	2	2		Marsden et al. (2009)	N/A
		Golden Jackal	*Canis aureus*	164	1	3	19.8	Stefanović et al. (2021)	N/A
		Grey Wolf	*Canis lupus*	175	1	11	22.8	Kennedy et al. (2007)	Kennedy et al. (2007)
Aves: Galliformes	Phasianidae	Chicken	*Gallus gallus*	21	1	1		Salomonsen et al. (2003)	N/A
Aves: Anseriformes	Anatidae	Duck	*Anas platyrhynchos*	9	1	4	0	Ren et al. (2011)	N/A
Aves: Procellariiformes	Hydrobatidae	Leach's Storm‐petrel	*Hydrobates leucorhous*	30	2	4	12.7	Rand et al. (2019)	Rand et al. (2019)
Aves: Ciconiiformes	Threskiornithidae	Japanese Crested Ibis	*Nipponia nippon*	5	3	2		Taniguchi et al. (2014)	Taniguchi et al. (2014)
Amphibia: Urodela	Cryptobranchidae	Chinese Giant Salamander	*Andrias davidianus*	8	2	15	3.7	Zhu et al. (2014)	Zhu et al. (2014)
Amphibia: Anura	Bufonidae	Australian Cane Toad	*Rhinella marina*	5	4	7	3.8	Lillie et al. (2016)	Lillie et al. (2016)
Actinopterygii: Acipenseriformes	Acipenseridae	Dabry's Sturgeon	*Acipenser dabryanus*	7	4	17	77.5	Chen et al. (2020)	Chen et al. (2020)
Actinopterygii: Anguilliformes	Anguillidae	European Eel	*Anguilla anguilla*	4	2	14	84.7	Bracamonte et al. (2015)	Bracamonte et al. (2015)
Actinopterygii: Siluriformes	Bagridae	Chinese Longsnout Catfish	*Leiocassis longirostris*	4	1	3	35.2	Shen et al. (2014)	Shen et al. (2011)
	Ictaluridae	Channel Catfish	*Ictalurus punctatus*	2	2	5	17.2	Godwin et al. (2000)	Godwin et al. (1997)
Actinopterygii: Salmoniformes	Salmonidae	Atlantic Salmon	*Salmo salar*	27	1	9	55.9	Gómez et al. (2010)	Gómez et al. (2010)
Actinopterygii: Cichliformes	Cichlidae	Nile Tilapia	*Oreochromis niloticus*	6	2	11	54.2	Pang et al. (2013)	Pang et al. (2013)
Actinopterygii: Perciformes	Sciaenidae	Large Yellow Croaker	*Pseudosciaena crocea*	2	1	3	4.3	Yu et al. (2010)	Yu et al. (2010)
		Miiuy Croaker	*Miichthys miiuy*	26	4	27	190.9	Xu et al. (2016)	Liu et al. (2016)
	Sparidae	Gilthead Seabream	*Sparus aurata*	2	3	9	87.1	Cuesta et al. (2006)	N/A
	Carangidae	Golden Pompano	*Trachinotus ovatus*	80	2	29	231.5	Cao et al. (2018)	N/A
Actinopterygii: Pleuronectiformes	Cynoglossidae	Half‐smooth Tongue Sole	*Cynoglossus semilaevis*	4	2	8	30.3	Xu et al. (2009)	Xu et al. (2009)
	Paralichthyidae	Japanese Flounder	*Paralichthys olivaceus*	212	2	55	256.9	Xu et al. (2010)	N/A
Actinopterygii: Synbranchiformes	Synbranchidae	Swamp Eel	*Monopterus albus*	5	3	5	8.7	Li, Sun, Meng, and Hong (2014)	Li, Sun, Hu, et al. (2014)

^a^Sample size.

^b^Number of MHC‐IIA genes assayed in the study.

^c^Number of unique MHC‐IIA exon 2 nucleotide sequences.

^d^Evidence for positive selection on a species' MHC‐IIA alleles.
