# Supplementary material for: Meta‐analysis of major histocompatibility complex (MHC) class IIA reveals polymorphism and positive selection in many vertebrate species
Source: Mol Ecol. 2022 Oct 19;31(24):6390–406. doi: 10.1111/mec.16726 (PMC9729452; doi:10.1111/mec.16726)
Supplement: Supplementary file 1 — Appendix S1 Supporting information [file MEC-31-6390-s001.docx]

##
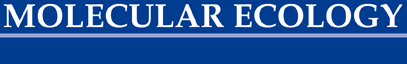
ONLINE SUPPORTING INFORMATION

## Meta-analysis of major histocompatibility complex (MHC) class IIA reveals polymorphism and positive selection in many vertebrate species

## Donald C Dearborn *^1,2^, Sophie Warren ^1,3^, and Frank Hailer *^4^

#### ^1^ Biology Department, Bates College, 44 Campus Ave, Lewiston, Maine, USA

#### ^2^ Roux Institute, Northeastern University, Fore St, Portland, Maine, USA

#### ^3^ Present address: Department of Health Policy, London School of Economics and Political Science, Houghton Street, London WC2A 2AE, UK

#### ^4^ Organisms and Environment, School of Biosciences, Cardiff University, Sir Martin Evans Building, Museum Avenue, Cardiff CF10 3AX, Wales, UK

#### * Co-corresponding authors: [ddearbor@bates.edu](mailto:ddearbor@bates.edu) and [HailerF@cardiff.ac.uk](mailto:HailerF@cardiff.ac.uk)

### 1. Details on Review of Sexual Selection or Disease Association Studies in MHC-IIA

Our search of the literature on mate choice and class II MHC yielded 116 papers that met our criteria, with the following results:

**106 papers with MHC-IIB data but not MHC-IIA data** (Aeschlimann et al., 2003; Agbali et al., 2010; Alcaide et al., 2012; Andreou et al., 2017; Bahr et al., 2012; Bahr & Wilson, 2011; Baratti et al., 2012; Bollmer et al., 2012; Bos et al., 2009; Buczek et al., 2016; Chuyanova et al., 2015; Cutrera et al., 2012; Cutrera et al., 2014; Dearborn et al., 2016; Dunn et al., 2013; Eizaguirre et al., 2011; Eizaguirre et al., 2009; Ekblom et al., 2010; Ekblom et al., 2004; Evans et al., 2012; Evans et al., 2013; Ferrandiz-Rovira et al., 2016; Forsberg et al., 2007; Freeman-Gallant et al., 2003; Gahr et al., 2018; Garamszegi et al., 2018; Garner et al., 2010; Gasparini et al., 2015; Gessner et al., 2017; Gillingham et al., 2009; Gohli et al., 2013; Grieves et al., 2019; Grogan et al., 2019; Häberli & Aeschlimann, 2004; Hale et al., 2009; Herdegen et al., 2013; Hoover et al., 2018; Huchard et al., 2013; Huchard et al., 2010; Ivy-Israel et al., 2021; Jacob et al., 2010; Jäger et al., 2007; Johansson et al., 2012; Juola & Dearborn, 2012; Kalbe et al., 2009; Knafler et al., 2012; Knapp et al., 2006; Kuduk et al., 2014; Kurtz et al., 2006; Landry et al., 2001; Leclaire et al., 2017; Leclaire et al., 2014; Lehnert et al., 2018; Lehnert et al., 2016; Lenz, Eizaguirre, et al., 2009; Lenz et al., 2018; Lenz et al., 2013; Liu et al., 2021; Løvlie et al., 2013; Matthews et al., 2010; McCairns et al., 2011; Meléndez-Rosa et al., 2018; Milinski et al., 2005; Mingju et al., 2021; Minias et al., 2020; Neff et al., 2008; Ottová et al., 2007; Pearson et al., 2018; Pearson et al., 2017; Pitcher & Neff, 2007; Promerová et al., 2013; Radwan et al., 2008; Reichard et al., 2012; Rekdal et al., 2019, 2021; Reusch et al., 2001; Roberts et al., 2006; Rymešová et al., 2017; Santos et al., 2016; Santos et al., 2017; Sauermann et al., 2001; Sawada et al., 2020; Schad et al., 2011; Schaschl et al., 2008; Schwensow, Eberle, et al., 2008; Schwensow, Fietz, et al., 2008; Setchell et al., 2013; Setchell et al., 2009, 2010; Silveira et al., 2020; Sin et al., 2015; Skarstein et al., 2005; Slade et al., 2016; Slade et al., 2019; Smith et al., 2018; Sommer, 2005; Sterck et al., 2017; Strandh et al., 2012; Sun et al., 2019; Thoß et al., 2011; Turner et al., 2009; Weir et al., 2012; Whittingham et al., 2015; Widdig et al., 2004; Winternitz et al., 2014; Yang et al., 2014),

**3 papers with MHC-IIA data but not MHC-IIB data** (Charbonnel et al., 2010; MacManes & Lacey, 2012; Tentelier et al., 2017), and

**7 papers with both IIA and IIB data** (Abts et al., 2018; Galaverni et al., 2016; Meléndez-Rosa et al., 2020; Promerová et al., 2017; Sommer et al., 2002; Yu et al., 2018; Zhang et al., 2020).

As summarized in the main manuscript, the presence of MHC-IIA data in sexual selection studies of class II MHC did not increase substantially as a function of year of publication (Table S1).

**Table S1. A generalized linear model of the presence/absence of MHC-IIA data in class II studies of sexual selection.** The GLM had a binomial error family with a logit link function. Explained deviance for the model was 3.9 %.

| **Term** | **Coefficient** | **SE** | **LRT**† | **d.f.** | **P** |
| --- | --- | --- | --- | --- | --- |
| Intercept | -234.19 | 150.98 |  |  |  |
| Year | 0.12 | 0.07 | 2.64 | 1 | 0.104 |

† X^2^ from Likelihood Ratio Test, implemented via single-term deletion

Our search of the literature on disease associations with class II MHC alleles yielded 95 papers that met our criteria, with the following results:

**78 papers with MHC-IIB data but not MHC-IIA data** (Acevedo-Whitehouse et al., 2018; André et al., 2017; Ariyanto et al., 2018; Arkush et al., 2002; Axtner & Sommer, 2012; Belasen et al., 2019; Biedrzycka & Kloch, 2016; Biedrzycka et al., 2011; Buczek et al., 2016; Cutrera et al., 2011, 2014; Dionne et al., 2009; Dionne et al., 2007; Ditchkoff et al., 2005; Du et al., 2011; Du et al., 2012; Dunn et al., 2013; Eizaguirre et al., 2012; Eizaguirre et al., 2011; Evans & Neff, 2009; Figueroa Castillo et al., 2011; Forletti et al., 2020; Fraser & Neff, 2010; Froeschke & Sommer, 2005, 2012; Garamszegi & Nunn, 2011; Garamszegi et al., 2015; Harf & Sommer, 2005; Hawley & Fleischer, 2012; Hu et al., 2017; Jin et al., 2010; Kloch et al., 2010; Kloch et al., 2013; Konnai et al., 2003; Kosch et al., 2016; Kurtz et al., 2004; Larruskain et al., 2010; Lei et al., 2016; Lenz, Wells, et al., 2009; Li et al., 2011; Lo et al., 2021; Loham et al., 2008; Lohm et al., 2002; Meyer-Lucht & Sommer, 2009; Molee et al., 2016; Montero et al., 2021; Natsopoulou et al., 2012; Oliver et al., 2009; Osborne et al., 2015; Osborne et al., 2017; Ottová et al., 2007; Radwan et al., 2010; Rakus et al., 2009; Rauch et al., 2006; Savage et al., 2019; Savage & Zamudio, 2016; Schad et al., 2005; Schwensow, Dausmann, et al., 2010; Schwensow, Eberle, et al., 2010; Schwensow et al., 2007; Seifertová et al., 2016; Šimková et al., 2006; Sin et al., 2014; Singh et al., 2012; Slade et al., 2017; Smith et al., 2011; Trujillo et al., 2021; Wedekind et al., 2004; Wegner et al., 2008; Wegner et al., 2003; Whittingham et al., 2018; Winternitz et al., 2014; Xu et al., 2008; J. Yang et al., 2016; M. Yang et al., 2016; Yu et al., 2014; Zhang & He, 2013; Zhu et al., 2018),

**9 papers with MHC-IIA data but not MHC-IIB data** (Chen et al., 2021; Deter et al., 2008; El-Magd et al., 2019; Hickford et al., 2011; Iacovakis et al., 2013; Kjøglum et al., 2006; Li et al., 2012; Liu et al., 2013; Wynne et al., 2007), and

**8 papers with both IIA and IIB data** (Grimholt et al., 2003; Guivier et al., 2010; Li et al., 2014; Miller et al., 2004; Soutter et al., 2018; Stear et al., 2019; Tollenaere et al., 2008; Zhang et al., 2015).

As summarized in the main manuscript, the presence of MHC-IIA data in class II disease association studies did not increase as a function of year of publication (Table S2).

**Table S2. A generalized linear model of the presence/absence of MHC-IIA data in class II studies of disease association.** The GLM had a binomial error family with a logit link function. Explained deviance for the model was less than 0.1 %.

| **Term** | **Coefficient** | **SE** | **LRT**† | **d.f.** | **P** |
| --- | --- | --- | --- | --- | --- |
| Intercept | -2.86 | 110.15 |  |  |  |
| Year | 0.001 | 0.055 | < 0.01 | 1 | 0.993 |

† X^2^ from Likelihood Ratio Test, implemented via single-term deletion

### 2. On Pooling Alleles Across Genes Within Species

In the Methods of the main manuscript, we outline the consideration we gave to issues involving the number and orthology of genes that were combined in a species’ dataset. The value of separately analyzing alleles by gene or lineage depends partly on the overall goal of the study. If the goal is to examine lineage evolution in deep evolutionary time or variation among genes in molecular evolution patterns, then examining each gene or lineage separately is vital. This is most easily envisioned with the well-characterized DPA, DQA, and DRA lineages in model mammalian systems. However, the main goal in our paper is to assess MHC variability in the context of fitness variation among individuals in a population, in which case consideration of diversity and selection on a species’ MHC-IIA alleles jointly (without respect to gene or lineage) seems a more suitable approach.

Beyond this conceptual distinction, though, is the issue of what is actually possible given the available information about gene identity and orthology in the existing MHC-IIA datasets. As we detail here, a large number of datasets are not amenable to ortholog-based assessments of diversity or selection. The 50 species with MHC-IIA datasets that met our inclusion criteria can be classified as follows:

- N=8 datasets with too few alleles for CodeML analysis of positive selection.
- N=14 datasets where the original paper assayed only 1 gene (5 DRA, 6 DQA, 3 unknown orthology), in which case our analyses necessarily did not pool alleles across genes.
- N=2 datasets consisting of alleles from multiple genes, assessed with separate amplification of each gene, but where orthology of those genes viz-a-viz other taxa was unknown.
- N=14 datasets where alleles could not be attributed to specific genes, because multiple genes were amplified by the same PCR primers.
- N=12 datasets with multiple genes of known orthology that were amplified with gene-specific primers. All were from mammals. Of these 12 datasets that would be amenable to subdivision into individual genes, for 6 of the 12 it would be impossible to run CodeML on at least one of the genes because that gene had too few alleles known/reported.

Another way to consider this issue of orthology would be to run a more focused analysis using only those species whose MHC-IIA datasets have sufficient data for CodeML analysis of all three mammalian MHC-IIA lineages (DPA, DQA, DRA). Unfortunately only two species in our datasets (cynomolgus macaque and pig-tailed macaque) meet this criteria. Two additional species (rhesus macaque and chimpanzee) have MHC-IIA sequences from all three gene lineages, but at least one lineage in each species has too few alleles to allow CodeML analysis.

Broadly, then, the 50 datasets vary widely in what is known about the gene(s) to which those sequences belong and the orthology of those genes, limiting our ability to effectively incorporate orthology into our analyses.

As described in the main manuscript and here in Table S5, the evidence for positive selection on MHC-IIA did not covary with the number of genes in the dataset, which might be interpreted to suggest that the necessary pooling across genes did not strongly influence our metrics of positive selection. Nonetheless, being able to fully account for gene identity, lineage history, and issues of linkage and genomic organization would greatly enhance our ability to explore patterns of polymorphism and selection in MHC-IIA. We look forward to a future in which genome-level understanding of MHC is sufficiently widespread for those explorations.

### 3. Supplementary Results on Allelic Diversity in MHC-IIA

In the main manuscript, we summarized the main findings of a GLM in which the number of MHC-IIA alleles reported in a species covaried positively with sample size and with number of genes assayed but did not differ between taxonomic groups (mammal, bird, amphibian, fish). Here in the Supporting Information we give more details on the parameters of that model (Table S3).

**Table S3. A generalized linear model explaining the number of MHC-IIA exon 2 nucleotide sequences across 50 species of mammals, birds, amphibians, and fish.** The number of alleles in a species covaried positively with sample size (NumAnimals) and number of genes assayed (NumGenes) but did not differ between mammals, birds, amphibians, and fish (Taxon). The GLM had a negative binomial error family with a log link function and dispersion parameter theta = 2.0. Explained deviance for the model was 30.8 %. See Figure 4 in the main manuscript for raw data and fitted values.

| **Term** | **Coefficient** | **SE** | **z** | **LRT**† | **d.f.** | **P** |
| --- | --- | --- | --- | --- | --- | --- |
| Intercept | 1.41 | 0.33 |  |  |  |  |
| NumAnimals | 2.75x10^-3^ | 8.31x10^-4^ |  | 11.1 | 1 | 0.001 |
| NumGenes | 0.34 | 0.14 |  | 6.3 | 1 | 0.012 |
| Taxon  reference level = Mammal |  |  |  | 6.5 | 3 | 0.088 |
| Bird | -1.04 | 0.49 | -2.11 |  | 1 | 0.035 |
| Amphibian | 0.08 | 0.58 | 0.14 |  | 1 | 0.892 |
| Fish | 0.31 | 0.26 | 1.17 |  | 1 | 0.241 |

† X^2^ from Likelihood Ratio Test, implemented via single-term deletion

The analysis detailed in Table S3, above, used MHC-IIA data from all 50 species and found no consistent differences in allelic diversity between mammals, birds, amphibians, and fish. Within those 50 datasets, there was enough representation from mammals (n=31) to test for finer-grained taxonomic variation in allelic diversity. Specifically, there were MHC-IIA datasets from six mammalian orders: Primates (n=5), Lagomorpha (n=2), Rodentia (n=4), Artiodactyla (n=10), Perissodactyla (n=3), and Carnivora (n=7). Thus, we explored this potential aspect of variation in the number of MHC-IIA alleles using a generalized linear model to test for main effects of the following fixed-effects predictor variables: sample size (NumAnimals; continuous), number of genes assayed (NumGenes; continuous; as determined by the original authors of each dataset), and mammalian order (Order; categorical: Artiodactyla, Carnivora, Lagomorpha, Perissodactyla, Primates, Rodentia). The number of unique nucleotide sequences (NumAllelesDNA) was modeled as a negative binomial GLM with a log link:

*NumAllelesDNA_i_ ~ NegBin (µ_i_, k)*

*E(NumAllelesDNA_i_) = µ_i_* and var*(NumAllelesDNA_i_) = µ_i_ + (µ_i_^2^ / k)*

*log (µ_i_) = η_i_*

*η_i_ = β_1_ + β_2_ x NumAnimals_i_ + β_3_ x NumGenes_i_ + β_4_ x Order_i_*

While controlling for potential effects of sample size and number of genes assayed, this model found comparatively high numbers of MHC-IIA alleles in the orders Lagomorpha, Primates, and Rodentia versus comparatively low numbers of alleles in the orders Carnivora, Artiodactyla, and Perissodactyla (Table S4, Fig. S1).

**Table S4. A generalized linear model explaining the number of MHC-IIA exon 2 nucleotide sequences across 31 species of mammals.** The number of alleles in a species differed between mammalian orders (Order) and covaried positively with sample size (NumAnimals) but not with number of genes assayed (NumGenes). The GLM had a negative binomial error family with a log link function and dispersion parameter theta = 4.8. Explained deviance for the model was 63.6 %. See Figure S1 for raw data and fitted values.

| **Term** | **Coefficient** | **SE** | **z** | **LRT**† | **d.f.** | **P** |
| --- | --- | --- | --- | --- | --- | --- |
| Intercept | 1.13 | 0.45 |  |  |  |  |
| NumAnimals | 1.52x10^-3^ | 7.17x10^-4^ |  | 4.3 | 1 | 0.038 |
| NumGenes | 0.26 | 0.21 |  | 1.8 | 1 | 0.182 |
| Order  reference level = Artiodactyla | |  |  | 38.8 | 5 | 0.001 |
| Carnivora | -0.18 | 0.31 | -0.56 |  | 1 | 0.574 |
| Lagomorpha | 1.61 | 0.46 | 3.51 |  | 1 | 0.001 |
| Perissodactyla | 0.12 | 0.44 | 0.28 |  | 1 | 0.778 |
| Primates | 1.19 | 0.36 | 3.31 |  | 1 | 0.001 |
| Rodentia | 0.88 | 0.32 | 2.68 |  | 1 | 0.007 |

† X^2^ from Likelihood Ratio Test, implemented via single-term deletion

**Figure S1.** **Raw data and GLM estimated marginal means for MHC-IIA allelic diversity across mammalian orders.** Observed values (green dots, n=31) and estimated marginal means (gray diamonds ± SE) from a GLM analyzing the number of MHC-IIA alleles (i.e. number of unique exon 2 nucleotide sequences) in 31 species of mammals. Estimated marginal means for each mammalian order account for effects of sample size and number of genes assayed. See Table S4 for GLM parameters. Points are horizontally dodged for clarity.

In addition to analyzing the number of MHC-IIA alleles, in the main manuscript we also reported the average within-species p-distance for those MHC-IIA alleles in 50 vertebrate species, using either nucleotide sequences or inferred amino acid sequences. Here, we show more detail, with boxplots of those two measures (Fig. S2).

**Figure S2**. **Distribution of mean p-distance values for MHC-IIA alleles within each of 50 vertebrate species.**

**A:** Nucleotide sequences.

**B:** Inferred amino acid sequences.

Because allele frequencies are unknown in most studies, p-distances for nucleotides and for amino acids were calculated with the unrealistic but necessary assumption that all alleles in a species have equal frequency. Species with a single allele were assigned a p-distance of zero. Line = median, box hinges = quartiles, whiskers = most extreme values not exceeding hinges ± 1.5 IQR.

Here we also show some additional detail on MHC-IIA polymorphism at the DNA and amino acid level (Fig. S3). For loci known to evolve under balancing selection such as MHC, one might expect a large number of amino acid sequences. Indeed, relative to the number of nucleotide sequences, the slope of the relationship was nearly 1 and the intercept did not differ significantly from zero (Fig. S3).

**Figure S3**. **Correlation of nucleotide and amino acid variability at MHC-IIA within species.** Number of MHC-IIA amino acid sequences within species nearly matched the corresponding number of nucleotide sequences in the species (linear regression, slope = 0.959 (95% CI: 0.926 to 0.991), intercept = -0.48 (95% CI: -1.01 to +0.06), r^2^ = 0.986, n = 50 species).

### 4. Supplementary Results on Positive Selection on MHC-IIA

In the main manuscript, we reported three measures of positive selection on MHC-IIA alleles in 42 vertebrate species: the log likelihood of model M8 over null model M7, the proportion of codons estimated as being under positive selection (i.e. p_1_ of M8), and the estimated value of ω_s_ of M8 (i.e. dN/dS for positively selected codons). Here, we show boxplots of those three measures (Fig. S4).

**Figure S4**. **Distribution of CodeML measures of positive selection on MHC-IIA alleles within each of 42 vertebrate species.**

**A:** Strength of evidence for positive selection, measured as log likelihood of model M8 over null model M7. Horizontal dashed line is critical value of log likelihood for rejecting M7, X^2^_df=2_ = 5.99.

**B:** Proportion of codons estimated as being under positive selection (i.e. p_1_ of M8).

**C:** Magnitude of positive selection (dN/dS, or ω_s_) at those positively selected codons within exon 2 of a given species. Line = median, box hinges = quartiles, whiskers = most extreme values not exceeding hinges ± 1.5 IQR.

In the main manuscript, we also reported that the strength of evidence for M8 in a species’ MHC-IIA alleles was modeled with a GLM, which found differences between taxonomic groups (mammal, bird, amphibian, fish) but no association with sample size or number of genes assayed. Here, we give more details on the parameters of that model (Table S5).

**Table S5. A generalized linear model explaining the interspecific variation in evidence for positive selection on a species’ set of MHC-IIA alleles.** The log likelihood support for positive selection in a species did not vary with sample size (NumAnimals) or number of genes assayed (NumGenes), but was greater in fish than in mammals, birds, or amphibians (Taxon). The GLM had a gamma error family with a log link function and dispersion parameter = 1.38. Explained deviance for the model was 27.5 %. See Figure 5 for raw data and estimated marginal means.

| **Term** | **Coefficient** | **SE** | **z** | **LRT**† | **d.f.** | **P** |
| --- | --- | --- | --- | --- | --- | --- |
| Intercept | 2.568 | 0.573 |  |  |  |  |
| NumAnimals | 1.76x10^-3^ | 1.40x10^-3^ |  | 1.72 | 1 | 0.1895 |
| NumGenes | 0.124 | 0.228 |  | 0.30 | 1 | 0.5840 |
| Taxon  reference level = Mammal |  |  |  | 19.94 | 3 | 0.0002 |
| Bird | -1.004 | 0.887 | -1.13 |  | 1 | 0.2650 |
| Amphibian | -1.599 | 0.902 | -1.77 |  | 1 | 0.0850 |
| Fish | 1.480 | 0.428 | 3.46 |  | 1 | 0.0014 |

† X^2^ from Likelihood Ratio Test, implemented via single-term deletion

### 5. Supplementary Results on Comparisons with MHC-IIB

In the main manuscript (top panel of Fig. 6), we summarized the primary findings of a GLMM that compared the number of alleles found in MHC-IIA versus MHC-IIB while controlling for potential effects of sample size, number of genes assayed, and taxonomic group. Here, we give more details on the parameters of that model (Table S6) and a graphical depiction of the marginal means by taxonomic group overlain with the difference between IIA and IIB (Fig. S5).

**Table S6. A generalized linear mixed model explaining the number of MHC class II exon 2 nucleotide sequences across 27 species, each with MHC-IIA and MHC-IIB datasets.** The number of alleles in a species was greater in MHC-IIB than in MHC-IIA when controlling for effects of sample size (NumAnimals), number of genes assayed (NumGenes), any differences between mammals, birds, amphibians, and fish (Taxon), and the dependencies inherent in having each species contribute both IIA and IIB data. The GLMM had a Poisson error family with a log link function and dispersion parameter theta = 0.75. The overall model had a pseudo-R^2^ of 0.52 for fixed effects (NumAnimals, NumGenes, and Taxon), and a total pseudo-R^2^ of 0.86 when including the random effect of Species. The intraclass correlation coefficient for Species was 0.20. See top panel of Figure 6 for raw data and estimated marginal means by Class, and Figure S5 for variation among taxonomic groups.

| **Term** | **Coefficient** | **SE** | **z** | **LRT**† | **d.f.** | **P** |
| --- | --- | --- | --- | --- | --- | --- |
| Intercept | 0.72 | 0.24 |  |  |  |  |
| MHC Class (reference level = IIA) | 0.29 | 0.09 |  | 10.9 | 1 | 0.0009 |
| NumAnimals | 0.014 | 0.0028 |  | 24.1 | 1 | 0.0001 |
| NumGenes | 0.18 | 0.04 |  | 20.1 | 1 | 0.0001 |
| Taxon  reference level = Mammal |  |  |  | 10.0 | 3 | 0.0183 |
| Bird | 0.23 | 0.46 | 0.50 |  | 1 | 0.6129 |
| Amphibian | 0.89 | 0.45 | 1.98 |  | 1 | 0.0479 |
| Fish | 0.85 | 0.27 | 3.10 |  | 1 | 0.0019 |

† X^2^ from Likelihood Ratio Test, implemented via single-term deletion

**Figure S5**. **GLMM estimated marginal means (± SE) for number of unique MHC class II exon 2 nucleotide sequences in a species.** Number of alleles was modeled as a function of fixed main effects of class (IIA, IIB), sample size, number of genes assayed, and taxonomic group (mammal, bird, amphibian, fish). Species was used as a random effect to account for dependencies in MHC-IIA and MHC-IIB data sets from the same species. Points are horizontally dodged for clarity. Estimated marginal means by class and taxonomic group account for effects of sample size and number of genes assayed. See Table S6 in this Supporting Information for model parameters and the top panel of Figure 6 in the main manuscript for the overall comparison between MHC-IIA and MHC-IIB.

In the main manuscript (bottom panel of Fig. 6), we summarized the primary findings of a GLMM that compared the evidence for positive selection in MHC-IIA versus MHC-IIB while controlling for potential effects of sample size, number of genes assayed, and taxonomic group (mammal, bird, amphibian, fish). Here, we give more details on the parameters of that model (Table S7) and a graphical depiction of the marginal means by taxonomic group overlain with the difference between IIA and IIB (Fig. S6).

**Table S7. A generalized linear mixed model explaining the interspecific variation in evidence for positive selection on a species’ set of MHC-IIA or MHC-IIB alleles.** The log likelihood support for positive selection in a species was greater in MHC-IIB than in MHC-IIA when controlling for effects of sample size (NumAnimals), number of genes assayed (NumGenes), differences between taxonomic groups (Taxon), and the potential dependencies inherent in having each species contribute both IIA and IIB data. The GLMM had a gamma error family with a log link function. Marginal and conditional pseudo-R^2^ for the model were both 0.63, because the random effect Species did not account for a measurable amount of variation in the response variable (in contrast to the GLMM of number of alleles, where Species was important). See bottom panel of Figure 6 for raw data and estimated marginal means by Class.

| **Term** | **Coefficient** | **SE** | **t** | **LRT**† | **d.f.** | **P** |
| --- | --- | --- | --- | --- | --- | --- |
| Intercept | 0.92 | 0.48 |  |  |  |  |
| Class (reference level = IIA) | 0.75 | 0.32 |  | 5.0 | 1 | 0.0254 |
| NumAnimals | 0.01 | 0.003 |  | 7.6 | 1 | 0.0057 |
| NumGenes | 0.36 | 0.10 |  | 12.8 | 1 | 0.0004 |
| Taxon  reference level = Mammal |  |  |  | 20.7 | 3 | 0.0001 |
| Bird | 1.38 | 0.73 | 1.90 |  | 1 | 0.0579 |
| Amphibian | -0.08 | 0.58 | -0.13 |  | 1 | 0.8944 |
| Fish | 1.87 | 0.40 | 4.62 |  | 1 | 0.0001 |

† X^2^ from Likelihood Ratio Test, implemented via single-term deletion

**Figure S6**. **GLMM estimated marginal means (± SE) for evidence of positive selection in a species, as measured by log likelihood of model M8 over M7.** Support for M8 was modeled as a function of fixed main effects of class (IIA, IIB), sample size, number of genes assayed, and taxonomic group (mammal, bird, amphibian, fish). Species was used as a random effect to account for potential dependencies in MHC-IIA and MHC-IIB data sets from the same species. Points are horizontally dodged for clarity. Estimated marginal means by class and taxonomic group account for effects of sample size and number of genes assayed. See Table S7 in this Supporting Information for model parameters and bottom panel of Figure 6 in the main manuscript for the overall comparison between MHC-IIA and MHC-IIB.

### 6. Data and R Code

The DNA sequence data were retrieved from published work, as listed in Appendix 1. Our single-species alignments, measures of polymorphism and positive selection, associated covariates, and R scripts for the GLMs and GLMMs are available on DataDryad [dataset] (Dearborn et al., 2022) at <https://doi.org/10.5061/dryad.fbg79cnx0>.

### REFERENCES

Abts, K. C., Ivy, J. A., & DeWoody, J. A. (2018). Demographic, environmental and genetic determinants of mating success in captive koalas (Phascolarctos cinereus). *Zoo Biology*, *37*(6), 416-433. <https://doi.org/10.1002/zoo.21457>

Acevedo-Whitehouse, K., Gulland, F. M. D., & Bowen, L. (2018). MHC class II DRB diversity predicts antigen recognition and is associated with disease severity in California sea lions naturally infected with Leptospira interrogans [Article]. *Infection, Genetics and Evolution*, *57*, 158-165. <https://doi.org/10.1016/j.meegid.2017.11.023>

Aeschlimann, P. B., Häberli, M. A., Reusch, T. B. H., Boehm, T., & Milinski, M. (2003). Female sticklebacks Gasterosteus aculeatus use self-reference to optimize MHC allele number during mate selection [Article]. *Behavioral Ecology and Sociobiology*, *54*(2), 119-126. <http://www.scopus.com/inward/record.url?eid=2-s2.0-0038824957&partnerID=40&md5=83ae24e49af8b68d93a17d39557b4886>

Agbali, M., Reichard, M., Bryjová, A., Bryja, J., & Smith, C. (2010). Mate choice for nonadditive genetic benefits correlate with mhc dissimilarity in the rose bitterling (*Rhodeus ocellatus*) [Article]. *Evolution*, *64*(6), 1683-1696. <https://doi.org/10.1111/j.1558-5646.2010.00961.x>

Alcaide, M., Rodríguez, A., Negro, J. J., & Serrano, D. (2012). Male transmission ratio distortion supports MHC-linked cryptic female choice in the lesser kestrel (Aves: Falconidae). *Behavioral Ecology and Sociobiology*, *66*(11), 1467-1473. <https://doi.org/10.1007/s00265-012-1401-9>

André, A., Millien, V., Galan, M., Ribas, A., & Michaux, J. R. (2017). Effects of parasite and historic driven selection on the diversity and structure of a MHC-II gene in a small mammal species (Peromyscus leucopus) undergoing range expansion [Article]. *Evolutionary Ecology*, *31*(5), 785-801. <https://doi.org/10.1007/s10682-017-9898-z>

Andreou, D., Eizaguirre, C., Boehm, T., & Milinski, M. (2017). Mate choice in sticklebacks reveals that immunogenes can drive ecological speciation. *Behavioral Ecology*, *28*(4), 953-961. <https://doi.org/10.1093/beheco/arx074>

Ariyanto, D., Carman, O., Soelistyowati, D. T., Zairin, M., Jr., & Syukur, M. (2018). MHC-II gene and its association with disease resistance to koi herpes virus in five strains of common carp [Article]. *AACL Bioflux*, *11*(5), 1564-1573.

Arkush, K. D., Giese, A. R., Mendonca, H. L., McBride, A. M., Marty, G. D., & Hedrick, P. W. (2002). Resistance to three pathogens in the endangered winter-run chinook salmon (Oncorhynchus tshawytscha): Effects of inbreeding and major histocompatibility complex genotypes. *Canadian Journal of Fisheries and Aquatic Sciences*, *59*(6), 966-975. <http://www.scopus.com/inward/record.url?eid=2-s2.0-0036381627&partnerID=40&md5=88d3db2249b5a08241550ae0aba80a12>

Axtner, J., & Sommer, S. (2012). The functional importance of sequence versus expression variability of MHC alleles in parasite resistance. *Genetica*, *140*(10-12), 407-420. <https://doi.org/10.1007/s10709-012-9689-y>

Bahr, A., Sommer, S., Mattle, B., & Wilson, A. B. (2012). Mutual mate choice in the potbellied seahorse (*Hippocampus abdominalis*) [Article]. *Behavioral Ecology*, *23*(4), 869-878. <https://doi.org/10.1093/beheco/ars045>

Bahr, A., & Wilson, A. B. (2011). The impact of sex-role reversal on the diversity of the major histocompatibility complex: Insights from the seahorse (Hippocampus abdominalis) [Article]. *BMC Evolutionary Biology*, *11*(1), Article 121. <https://doi.org/10.1186/1471-2148-11-121>

Baratti, M., Dessì-Fulgheri, F., Ambrosini, R., Bonisoli-Alquati, A., Caprioli, M., Goti, E., Matteo, A., Monnanni, R., Ragionieri, L., Ristori, E., Romano, M., Rubolini, D., Scialpi, A., & Saino, N. (2012). MHC genotype predicts mate choice in the ring-necked pheasant *Phasianus colchicus*. *Journal of Evolutionary Biology*, *25*(8), 1531-1542. <http://www.scopus.com/inward/record.url?eid=2-s2.0-84864007195&partnerID=40&md5=aa9534dcb53e0be77d3a1719d334649b>

Belasen, A. M., Bletz, M. C., Leite, D. S., Toledo, L. F., & James, T. Y. (2019). Long-term habitat fragmentation is associated with reduced MHC IIB diversity and increased infections in amphibian hosts [Article]. *Frontiers in Ecology and Evolution*, *6*(JAN), Article 236. <https://doi.org/10.3389/fevo.2018.00236>

Biedrzycka, A., & Kloch, A. (2016). Development of novel associations between MHC alleles and susceptibility to parasitic infections in an isolated population of an endangered mammal [Article]. *Infection, Genetics and Evolution*, *44*, 210-217. <https://doi.org/10.1016/j.meegid.2016.07.014>

Biedrzycka, A., Kloch, A., Buczek, M., & Radwan, J. (2011). Major histocompatibility complex DRB genes and blood parasite loads in fragmented populations of the spotted suslik Spermophilus suslicus [Article]. *Mammalian Biology*, *76*(6), 672-677. <https://doi.org/10.1016/j.mambio.2011.05.002>

Bollmer, J. L., Dunn, P. O., Freeman-Gallant, C. R., & Whittingham, L. A. (2012). Social and extra-pair mating in relation to major histocompatibility complex variation in common yellowthroats. *Proceedings of the Royal Society B: Biological Sciences*, *279*(1748), 4778-4785. <http://www.scopus.com/inward/record.url?eid=2-s2.0-84868097945&partnerID=40&md5=87a1179bfdd202af43969f16ba6b3ae0>

Bos, D. H., Williams, R. N., Gopurenko, D., Bulut, Z., & Dewoody, J. A. (2009). Condition-dependent mate choice and a reproductive disadvantage for MHC-divergent male tiger salamanders [Article]. *Molecular Ecology*, *18*(15), 3307-3315. <https://doi.org/10.1111/j.1365-294X.2009.04242.x>

Buczek, M., Okarma, H., Demiaszkiewicz, A. W., & Radwan, J. (2016). MHC, parasites and antler development in red deer: No support for the Hamilton & Zuk hypothesis [Article]. *Journal of Evolutionary Biology*, *29*(3), 617-632. <https://doi.org/10.1111/jeb.12811>

Charbonnel, N., Bryja, J., Galan, M., Deter, J., Tollenaere, C., Chaval, Y., Morand, S., & Cosson, J. (2010). Negative relationships between cellular immune response, Mhc class II heterozygosity and secondary sexual trait in the montane water vole. *Evolutionary Applications*, *3*(3), 279-290. <https://doi.org/10.1111/j.1752-4571.2009.00108.x>

Chen, J., Zheng, Y., Zhi, T., Xu, X., Zhang, S., Brown, C. L., & Yang, T. (2021). MHC II α polymorphism of Nile tilapia, Oreochromis niloticus, and its association with the susceptibility to Gyrodactylus cichlidarum (Monogenea) infection [Article]. *Aquaculture*, *539*, Article 736637. <https://doi.org/10.1016/j.aquaculture.2021.736637>

Chuyanova, A. A., Tsepokina, A. V., Shabaldin, A. V., Litvinova, N. A., Zubrilova, K. Y., & Boldyreva, M. N. (2015). Features olfactory screening for HLA-DRB1 among unrelated donors of different sex. *Immunologiya*, *36*(2), 90-95. <https://www.scopus.com/inward/record.uri?eid=2-s2.0-84941560177&partnerID=40&md5=c8126e3cfccb1729fdb3d4fe4fef4719>

Cutrera, A. P., Fanjul, M. S., & Zenuto, R. R. (2012). Females prefer good genes: MHC-associated mate choice in wild and captive tuco-tucos [Article]. *Animal Behaviour*, *83*(3), 847-856. <https://doi.org/10.1016/j.anbehav.2012.01.006>

Cutrera, A. P., Zenuto, R. R., & Lacey, E. A. (2011). MHC variation, multiple simultaneous infections and physiological condition in the subterranean rodent Ctenomys talarum. *Infection, Genetics and Evolution*, *11*(5), 1023-1036. <https://doi.org/10.1016/j.meegid.2011.03.016>

Cutrera, A. P., Zenuto, R. R., & Lacey, E. A. (2014). Interpopulation differences in parasite load and variable selective pressures on MHC genes in Ctenomys talarum. *Journal of Mammalogy*, *95*(4), 679-695. <https://doi.org/10.1644/13-MAMM-A-120>

Dearborn, D. C., Gager, A. B., McArthur, A. G., Gilmour, M. E., Mandzhukova, E., & Mauck, R. A. (2016). Gene duplication and divergence produce divergent MHC genotypes without disassortative mating [Article]. *Molecular Ecology*, *25*(17), 4355-4367. <https://doi.org/10.1111/mec.13747>

Dearborn, D. C., Warren, S., & Hailer, F. (2022). *Polymorphism and selection in major histocompatibility complex (MHC) class IIA* <https://doi.org/https://doi.org/10.5061/dryad.fbg79cnx0>

Deter, J., Bryja, J., Chaval, Y., Galan, M., Henttonen, H., Laakkonen, J., Voutilainen, L., Vapalahti, O., Vaheri, A., Salvador, A. R., Morand, S., Cosson, J. F., & Charbonnel, N. (2008). Association between the DQA MHC class II gene and Puumala virus infection in Myodes glareolus, the bank vole. *Infection, Genetics and Evolution*, *8*(4), 450-458. <https://doi.org/10.1016/j.meegid.2007.07.003>

Dionne, M., Miller, K. M., Dodson, J. J., & Bernatchez, L. (2009). MHC standing genetic variation and pathogen resistance in wild Atlantic salmon. *Philosophical Transactions of the Royal Society B: Biological Sciences*, *364*(1523), 1555-1565. <https://doi.org/10.1098/rstb.2009.0011>

Dionne, M., Miller, K. M., Dodson, J. J., Caron, F., & Bernatchez, L. (2007). Clinal variation in MHC diversity with temperature: Evidence for the role of host-pathogen interaction on local adaptation in Atlantic salmon [Article]. *Evolution*, *61*(9), 2154-2164. <https://doi.org/10.1111/j.1558-5646.2007.00178.x>

Ditchkoff, S. S., Hoofer, S. R., Lochmiller, R. L., Masters, R. E., & Van Den Bussche, R. A. (2005). MHC-DRB evolution provides insight into parasite resistance in white-tailed deer [Article]. *Southwestern Naturalist*, *50*(1), 57-64. <https://doi.org/10.1894/0038-4909(2005)050><0057:MEPIIP>2.0.CO;2

Du, M., Chen, S. L., Liu, Y. H., Liu, Y., & Yang, J. F. (2011). MHC polymorphism and disease resistance to vibrio anguillarum in 8 families of half-smooth tongue sole (Cynoglossus semilaevis). *BMC Genetics*, *12*, Article 78. <https://doi.org/10.1186/1471-2156-12-78>

Du, M., Chen, S. L., Liu, Y. H., Niu, B. Z., Yang, J. F., & Zhang, B. (2012). MHC polymorphism and disease-resistance to Edwardsiella tarda in six turbot (Scophthalmus maximus) families [Article]. *Chinese Science Bulletin*, *57*(25), 3262-3269. <https://doi.org/10.1007/s11434-012-5179-y>

Dunn, P. O., Bollmer, J. L., Freeman-Gallant, C. R., & Whittingham, L. A. (2013). Mhc variation is related to a sexually selected ornament, survival, and parasite resistance in common yellowthroats. *Evolution*, *67*(3), 679-687. <https://doi.org/10.1111/j.1558-5646.2012.01799.x>

Eizaguirre, C., Lenz, T. L., Kalbe, M., & Milinski, M. (2012). Rapid and adaptive evolution of MHC genes under parasite selection in experimental vertebrate populations [Article]. *Nature Communications*, *3*, Article 621. <https://doi.org/10.1038/ncomms1632>

Eizaguirre, C., Lenz, T. L., Sommerfeld, R. D., Harrod, C., Kalbe, M., & Milinski, M. (2011). Parasite diversity, patterns of MHC II variation and olfactory based mate choice in diverging three-spined stickleback ecotypes. *Evolutionary Ecology*, *25*(3), 605-622. <https://doi.org/10.1007/s10682-010-9424-z>

Eizaguirre, C., Yeates, S. E., Lenz, T. L., Kalbe, M., & Milinski, M. (2009). MHC-based mate choice combines good genes and maintenance of MHC polymorphism. *Molecular Ecology*, *18*(15), 3316-3329. <http://www.scopus.com/inward/record.url?eid=2-s2.0-68149147158&partnerID=40&md5=0ef2706b49262bc58285ec52204c57a3>

Ekblom, R., Saether, S. A., Fiske, P., Käläs, J. A., & Höglund, J. (2010). Balancing selection, sexual selection and geographic structure in MHC genes of Great Snipe. *Genetica*, *138*(4), 453-461. <http://www.scopus.com/inward/record.url?eid=2-s2.0-77949289835&partnerID=40&md5=f0ec7389f16f220a9e37f04e64499680>

Ekblom, R., Saether, S. A., Grahn, M., Fiske, P., Kålås, J. A., & Höglund, J. (2004). Major histocompatibility complex variation and mate choice in a lekking bird, the great snipe (*Gallinago media*). *Molecular Ecology*, *13*(12), 3821-3828. <http://www.scopus.com/inward/record.url?eid=2-s2.0-9644279499&partnerID=40&md5=2d793ee4741958157f55c4140a28bec6>

El-Magd, M. A., El-Said, K. S., El-Semlawy, A. A., Tanekhy, M., Afifi, M., & Mohamed, T. M. (2019). Association of MHC IIA polymorphisms with disease resistance in Aeromonas hydrophila-challenged Nile tilapia [Article]. *Developmental and Comparative Immunology*, *96*, 126-134. <https://doi.org/10.1016/j.dci.2019.03.002>

Evans, M. L., Dionne, M., Miller, K. M., & Bernatchez, L. (2012). Mate choice for major histocompatibility complex genetic divergence as a bet-hedging strategy in the atlantic salmon (*Salmo salar*) [Article]. *Proceedings of the Royal Society B: Biological Sciences*, *279*(1727), 379-386. <https://doi.org/10.1098/rspb.2011.0909>

Evans, M. L., & Neff, B. D. (2009). Major histocompatibility complex heterozygote advantage and widespread bacterial infections in populations of Chinook salmon (Oncorhynchus tshawytscha). *Molecular Ecology*, *18*(22), 4716-4729. <https://doi.org/10.1111/j.1365-294X.2009.04374.x>

Evans, M. L., Neff, B. D., & Heath, D. D. (2013). Behavioural and genetic analyses of mate choice and reproductive success in two Chinook salmon populations [Article]. *Canadian Journal of Fisheries and Aquatic Sciences*, *70*(2), 263-270. <https://doi.org/10.1139/cjfas-2012-0415>

Ferrandiz-Rovira, M., Allainé, D., Callait-Cardinal, M. P., & Cohas, A. (2016). Mate choice for neutral and MHC genetic characteristics in Alpine marmots: different targets in different contexts? *Ecology and Evolution*, *6*(13), 4243-4257. <https://doi.org/10.1002/ece3.2189>

Figueroa Castillo, J. A., Medina, R. D. M., Villalobos, J. M. B., Gayosso-Vázquez, A., Ulloa-Arvízu, R., Rodríguez, R. A., Ramírez, H. P., & Alonso Morales, R. A. (2011). Association between major histocompatibility complex microsatellites, fecal egg count, blood packed cell volume and blood eosinophilia in Pelibuey sheep infected with Haemonchus contortus [Article]. *Veterinary Parasitology*, *177*(3-4), 339-344. <https://doi.org/10.1016/j.vetpar.2010.11.056>

Forletti, A., Lützelschwab, C. M., Cepeda, R., Esteban, E. N., & Gutiérrez, S. E. (2020). Early events following bovine leukaemia virus infection in calves with different alleles of the major histocompatibility complex DRB3 gene [Article]. *Veterinary Research*, *51*(1), Article 4. <https://doi.org/10.1186/s13567-019-0732-1>

Forsberg, L. A., Dannewitz, J., Petersson, E., & Grahn, M. (2007). Influence of genetic dissimilarity in the reproductive success and mate choice of brown trout - Females fishing for optimal MHC dissimilarity. *Journal of Evolutionary Biology*, *20*(5), 1859-1869. <http://www.scopus.com/inward/record.url?eid=2-s2.0-34547890262&partnerID=40&md5=19f5b0d9cd6d4f199ef0b48c61fe348d>

Fraser, B. A., & Neff, B. D. (2010). Parasite mediated homogenizing selection at the MHC in guppies [Article]. *Genetica*, *138*(2), 273-278. <https://doi.org/10.1007/s10709-009-9402-y>

Freeman-Gallant, C. R., Meguerdichian, M., Wheelwright, N. T., & Sollecito, S. V. (2003). Social pairing and female mating fidelity predicted by restriction fragment length polymorphism similarity at the major histocompatibility complex in a songbird. *Molecular Ecology*, *12*(11), 3077-3083. <http://www.scopus.com/inward/record.url?eid=2-s2.0-0242540541&partnerID=40&md5=24dbdc8fc36cee2c8b0dbb700ef9b9a6>

Froeschke, G., & Sommer, S. (2005). MHC class II DRB variability and parasite load in the striped mouse (Rhabdomys pumilio) in the Southern Kalahari. *Molecular Biology and Evolution*, *22*(5), 1254-1259. <https://doi.org/10.1093/molbev/msi112>

Froeschke, G., & Sommer, S. (2012). Insights into the complex associations between MHC class II DRB polymorphism and multiple gastrointestinal parasite infestations in the striped mouse. *Plos One*, *7*(2), Article e31820. <https://doi.org/10.1371/journal.pone.0031820>

Gahr, C. L., Boehm, T., & Milinski, M. (2018). Female assortative mate choice functionally validates synthesized male odours of evolving stickleback river-lake ecotypes. *Biology Letters*, *14*(12), Article 20180730. <https://doi.org/10.1098/rsbl.2018.0730>

Galaverni, M., Caniglia, R., Milanesi, P., Lapalombella, S., Fabbri, E., & Randi, E. (2016). Choosy Wolves? Heterozygote Advantage but No Evidence of MHC-Based Disassortative Mating. *Journal of Heredity*, *107*(2), 134-142. <https://doi.org/10.1093/jhered/esv090>

Garamszegi, L. Z., & Nunn, C. L. (2011). Parasite-mediated evolution of the functional part of the MHC in primates [Article]. *Journal of Evolutionary Biology*, *24*(1), 184-195. <https://doi.org/10.1111/j.1420-9101.2010.02156.x>

Garamszegi, L. Z., Zagalska-Neubauer, M., Canal, D., Blázi, G., Laczi, M., Nagy, G., Szöllősi, E., Vaskuti, É., Török, J., & Zsebők, S. (2018). MHC-mediated sexual selection on birdsong: Generic polymorphism, particular alleles and acoustic signals. *Molecular Ecology*, *27*(11), 2620-2633. <https://doi.org/10.1111/mec.14703>

Garamszegi, L. Z., Zagalska-Neubauer, M., Canal, D., Markó, G., Szász, E., Zsebok, S., Szöllosi, E., Herczeg, G., & Török, J. (2015). Malaria parasites, immune challenge, MHC variability, and predator avoidance in a passerine bird [Article]. *Behavioral Ecology*, *26*(5), 1292-1302. <https://doi.org/10.1093/beheco/arv077>

Garner, S. R., Bortoluzzi, R. N., Heath, D. D., & Neff, B. D. (2010). Sexual conflict inhibits female mate choice for major histocompatibility complex dissimilarity in Chinook salmon <http://rspb.royalsocietypublishing.org/> subscriptions. *Proceedings of the Royal Society B: Biological Sciences*, *277*(1683), 885-894. <https://doi.org/10.1098/rspb.2009.1639>

Gasparini, C., Congiu, L., & Pilastro, A. (2015). Major histocompatibility complex similarity and sexual selection: Different does not always mean attractive. *Molecular Ecology*, *24*(16), 4286-4295. <https://doi.org/10.1111/mec.13222>

Gessner, C., Nakagawa, S., Zavodna, M., & Gemmell, N. J. (2017). Sexual selection for genetic compatibility: The role of the major histocompatibility complex on cryptic female choice in Chinook salmon (Oncorhynchus tshawytscha). *Heredity*, *118*(5), 442-452. <https://doi.org/10.1038/hdy.2016.116>

Gillingham, M. A. F., Richardson, D. S., Løvlie, H., Moynihan, A., Worley, K., & Pizzari, T. (2009). Cryptic preference for MHC-dissimilar females in male red junglefowl, *Gallus gallus*. *Proceedings of the Royal Society B: Biological Sciences*, *276*(1659), 1083-1092. <http://www.scopus.com/inward/record.url?eid=2-s2.0-60049097620&partnerID=40&md5=b82ca5abb29c2904ebb458603b32726e>

Gohli, J., Anmarkrud, J. A., Johnsen, A., Kleven, O., Borge, T., & Lifjeld, J. T. (2013). Female promiscuity is positively associated with neutral and selected genetic diversity in passerine birds. *Evolution*, *67*(5), 1406-1419. <https://doi.org/10.1111/evo.12045>

Grieves, L. A., Gloor, G. B., Bernards, M. A., & MacDougall-Shackleton, E. A. (2019). Songbirds show odour-based discrimination of similarity and diversity at the major histocompatibility complex. *Animal Behaviour*, *158*, 131-138. <https://doi.org/10.1016/j.anbehav.2019.10.005>

Grimholt, U., Larsen, S., Nordmo, R., Midtlyng, P., Kjoeglum, S., Storset, A., Saebø, S., & Stet, R. J. M. (2003). MHC polymorphism and disease resistance in Atlantic salmon (Salmo salar); facing pathogens with single expressed major histocompatibility class I and class II loci [Article]. *Immunogenetics*, *55*(4), 210-219. <https://doi.org/10.1007/s00251-003-0567-8>

Grogan, K. E., Harris, R. L., Boulet, M., & Drea, C. M. (2019). Genetic variation at MHC class II loci influences both olfactory signals and scent discrimination in ring-tailed lemurs [Article]. *BMC Evolutionary Biology*, *19*(1), Article 171. <https://doi.org/10.1186/s12862-019-1486-0>

Guivier, E., Galan, M., Malé, P. J. G., Kallio, E. R., Voutilainen, L., Henttonen, H., Olsson, G. E., Lundkvist, Å., Tersago, K., Augot, D., Cosson, J. F., & Charbonnel, N. (2010). Associations between MHC genes and Puumala virus infection in Myodes glareolus are detected in wild populations, but not from experimental infection data. *Journal of General Virology*, *91*(10), 2507-2512. <https://doi.org/10.1099/vir.0.021600-0>

Häberli, M. A., & Aeschlimann, P. B. (2004). Male traits influence odour-based mate choice in the three-spined stickleback. *Journal of Fish Biology*, *64*(3), 702-710. <https://doi.org/10.1111/j.1095-8649.2004.00338.x>

Hale, M. L., Verduijn, M. H., Møller, A. P., Wolff, K., & Petrie, M. (2009). Is the peacock's train an honest signal of genetic quality at the major histocompatibility complex? *Journal of Evolutionary Biology*, *22*(6), 1284-1294. <https://doi.org/10.1111/j.1420-9101.2009.01746.x>

Harf, R., & Sommer, S. (2005). Association between major histocompatibility complex class II DRB alleles and parasite load in the hairy-footed gerbil, Gerbillurus paeba, in the southern Kalahari. *Molecular Ecology*, *14*(1), 85-91. <https://doi.org/10.1111/j.1365-294X.2004.02402.x>

Hawley, D. M., & Fleischer, R. C. (2012). Contrasting epidemic histories reveal pathogen-mediated balancing selection on class II MHC diversity in a wild songbird [Article]. *Plos One*, *7*(1), Article e30222. <https://doi.org/10.1371/journal.pone.0030222>

Herdegen, M., Nadachowska-Brzyska, K., Konowalik, A., Babik, W., & Radwan, J. (2013). Heterozygosity, sexual ornament and body size in the crested newt. *Journal of Zoology*, *291*(2), 146-153. <https://doi.org/10.1111/jzo.12056>

Hickford, J. G. H., Forrest, R. H. J., Zhou, H., Fang, Q., & Frampton, C. M. (2011). Association between variation in faecal egg count for a mixed field-challenge of nematode parasites and ovine MHC-DQA2 polymorphism [Article]. *Veterinary Immunology and Immunopathology*, *144*(3-4), 312-320. <https://doi.org/10.1016/j.vetimm.2011.08.014>

Hoover, B., Alcaide, M., Jennings, S., Sin, S. Y. W., Edwards, S. V., & Nevitt, G. A. (2018). Ecology can inform genetics: Disassortative mating contributes to MHC polymorphism in Leach’s storm-petrels (*Oceanodroma leucorhoa*) [Article]. *Molecular Ecology*, *27*(16), 3371-3385. <https://doi.org/10.1111/mec.14801>

Hu, W., Dong, B., Kong, S., Mao, Y., & Zheng, R. (2017). Pathogen resistance and gene frequency stability of major histocompatibility complex class IIB alleles in the giant spiny frog Quasipaa spinosa [Article]. *Aquaculture*, *468*, 410-416. <https://doi.org/10.1016/j.aquaculture.2016.11.001>

Huchard, E., Baniel, A., Schliehe-Diecks, S., & Kappeler, P. M. (2013). MHC-disassortative mate choice and inbreeding avoidance in a solitary primate. *Molecular Ecology*, *22*(15), 4071-4086. <https://doi.org/10.1111/mec.12349>

Huchard, E., Knapp, L. A., Wang, J., Raymond, M., & Cowlishaw, G. (2010). MHC, mate choice and heterozygote advantage in a wild social primate. *Molecular Ecology*, *19*(12), 2545-2561. <http://www.scopus.com/inward/record.url?eid=2-s2.0-77953524800&partnerID=40&md5=0b76fc92f4876524b16852493ccfcf64>

Iacovakis, C., Mamuris, Z., Moutou, K. A., Touloudi, A., Hammer, A. S., Valiakos, G., Giannoulis, T., Stamatis, C., Spyrou, V., Athanasiou, L. V., Kantere, M., Asferg, T., Giannakopoulos, A., Salomonsen, C. M., Bogdanos, D., Birtsas, P., Petrovska, L., Hannant, D., & Billinis, C. (2013). Polarisation of Major Histocompatibility Complex II Host Genotype with Pathogenesis of European Brown Hare Syndrome Virus. *Plos One*, *8*(9), Article e74360. <https://doi.org/10.1371/journal.pone.0074360>

Ivy-Israel, N. M. D., Moore, C. E., Schwartz, T. S., Steury, T. D., Zohdy, S., Newbolt, C. H., & Ditchkoff, S. S. (2021). Association between sexually selected traits and allelic distance in two unlinked MHC II loci in white-tailed deer (Odocoileus virginianus). *Evolutionary Ecology*, *35*(3), 513-535. <https://doi.org/10.1007/s10682-021-10108-x>

Jacob, A., Evanno, G., Von Siebenthal, B. A., Grossen, C., & Wedekind, C. (2010). Effects of different mating scenarios on embryo viability in brown trout. *Molecular Ecology*, *19*(23), 5296-5307. <https://doi.org/10.1111/j.1365-294X.2010.04884.x>

Jäger, I., Eizaguirre, C., Griffiths, S. W., Kalbe, M., Krobbach, C. K., Reusch, T. B. H., Schaschl, H., & Milinski, M. (2007). Individual MHC class I and MHC class IIB diversities are associated with male and female reproductive traits in the three-spined stickleback. *Journal of Evolutionary Biology*, *20*(5), 2005-2015. <http://www.scopus.com/inward/record.url?eid=2-s2.0-34547891023&partnerID=40&md5=d5b9dfb44b4bce4b6311fa53877aa2fc>

Jin, Y. C., Wei, P., Wei, X. X., Zhao, Z. Y., & Li, Y. (2010). Marek's disease resistant/susceptible MHC haplotypes in Xiayan chickens identified on the basis of BLB2 PCR-RFLP and BLB2/BF2 sequence analyses [Article]. *British Poultry Science*, *51*(4), 530-539. <https://doi.org/10.1080/00071668.2010.508489>

Johansson, M. L., Clifford, K., Fodness, B., Vazquez, N. A., & Banks, M. A. (2012). Mate selection in captive-breeding rockfishes Sebastes spp.: Inference from parentage analysis and the major histocompatibility complex (MHC). *Marine Ecology Progress Series*, *460*, 195-206. <https://doi.org/10.3354/meps09803>

Juola, F. A., & Dearborn, D. C. (2012). Sequence-based evidence for major histocompatibility complex-disassortative mating in a colonial seabird. *Proceedings of the Royal Society B: Biological Sciences*, *279*(1726), 153-162. <http://www.scopus.com/inward/record.url?eid=2-s2.0-81855196017&partnerID=40&md5=3e5c7f62934387423e09b93290959fc7>

Kalbe, M., Eizaguirre, C., Dankert, I., Reusch, T. B., Sommerfeld, R. D., Wegner, K. M., & Milinski, M. (2009). Lifetime reproductive success is maximized with optimal major histocompatibility complex diversity. *Proceedings of the Royal Society B: Biological Sciences*, *276*(1658), 925-934. <http://www.scopus.com/inward/record.url?eid=2-s2.0-63849093692&partnerID=40&md5=a3726b92a21b115bde439a00bae8be42>

Kjøglum, S., Larsen, S., Bakke, H. G., & Grimholt, U. (2006). How specific MHC class I and class II combinations affect disease resistance against infectious salmon anaemia in Atlantic salmon (Salmo salar) [Article]. *Fish and Shellfish Immunology*, *21*(4), 431-441. <https://doi.org/10.1016/j.fsi.2006.02.001>

Kloch, A., Babik, W., Bajer, A., Siński, E., & Radwan, J. (2010). Effects of an MHC-DRB genotype and allele number on the load of gut parasites in the bank vole Myodes glareolus. *Molecular Ecology*, *19*(SUPPL. 1), 255-265. <https://doi.org/10.1111/j.1365-294X.2009.04476.x>

Kloch, A., Baran, K., Buczek, M., Konarzewski, M., & Radwan, J. (2013). MHC influences infection with parasites and winter survival in the root vole *Microtus oeconomus*. *Evolutionary Ecology*, *27*(3), 635-653. <http://www.scopus.com/inward/record.url?eid=2-s2.0-84875393211&partnerID=40&md5=5919cb0cf81bab21aabf89b65413af70>

Knafler, G. J., Clark, J. A., Boersma, P. D., & Bouzat, J. L. (2012). MHC diversity and mate choice in the magellanic penguin, *Spheniscus magellanicus*. *Journal of Heredity*, *103*(6), 759-768. <https://doi.org/10.1093/jhered/ess054>

Knapp, L. A., Robson, J., & Waterhouse, J. S. (2006). Olfactory signals and the MHC: A review and a case study in Lemur catta. American Journal of Primatology,

Konnai, S., Takeshima, S. N., Tajima, S., Yin, S. A., Okada, K., Onuma, M., & Aida, Y. (2003). The influence of ovine MHC class II DRB1 alleles on immune response in bovine leukemia virus infection [Article]. *Microbiology and Immunology*, *47*(3), 223-232. <https://doi.org/10.1111/j.1348-0421.2003.tb03391.x>

Kosch, T. A., Bataille, A., Didinger, C., Eimes, J. A., Rodríguez-Brenes, S., Ryan, M. J., & Waldman, B. (2016). Major histocompatibility complex selection dynamics in pathogen-infected túngara frog (Physalaemus pustulosus) populations [Article]. *Biology Letters*, *12*(8), Article 20160345. <https://doi.org/10.1098/rsbl.2016.0345>

Kuduk, K., Babik, W., Bellemain, E., Valentini, A., Zedrosser, A., Taberlet, P., Kindberg, J., Swenson, J. E., & Radwan, J. (2014). No evidence for the effect of MHC on male mating success in the brown bear. *Plos One*, *9*(12), Article e113414. <https://doi.org/10.1371/journal.pone.0113414>

Kurtz, J., Kalbe, M., Aeschlimann, P. B., Haberli, M. A., Wegner, K. M., Reusch, T. B. H., & Milinski, M. (2004). Major histocompatibility complex diversity influences parasite resistance and innate immunity in sticklebacks. *Proceedings of the Royal Society of London Series B-Biological Sciences*, *271*(1535), 197-204. <Go to ISI>://000188171300013

Kurtz, J., Wegner, K. M., Kalbe, M., Reusch, T. B. H., Schaschl, H., Hasselquist, D., & Milinski, M. (2006). MHC genes and oxidative stress in sticklebacks: An immuno-ecological approach. *Proceedings of the Royal Society B: Biological Sciences*, *273*(1592), 1407-1414. <https://doi.org/10.1098/rspb.2005.3450>

Landry, C., Garant, D., Duchesne, P., & Bernatchez, L. (2001). 'Good genes as heterozygosity': The major histocompatibility complex and mate choice in Atlantic salmon (*Salmo salar*). *Proceedings of the Royal Society B: Biological Sciences*, *268*(1473), 1279-1285. <http://www.scopus.com/inward/record.url?eid=2-s2.0-0035933288&partnerID=40&md5=e1ca069d98c680101e3f1de6686b1295>

Larruskain, A., Minguijón, E., García-Etxebarria, K., Moreno, B., Arostegui, I., Juste, R. A., & Jugo, B. M. (2010). MHC class II DRB1 gene polymorphism in the pathogenesis of Maedi-Visna and pulmonary adenocarcinoma viral diseases in sheep [Article]. *Immunogenetics*, *62*(2), 75-83. <https://doi.org/10.1007/s00251-009-0419-2>

Leclaire, S., Strandh, M., Mardon, J., Westerdahl, H., & Bonadonna, F. (2017). Odour-based discrimination of similarity at the major histocompatibility complex in birds. *Proceedings of the Royal Society B: Biological Sciences*, *284*(1846), Article 20162466. <https://doi.org/10.1098/rspb.2016.2466>

Leclaire, S., van Dongen, W. F. D., Voccia, S., Merkling, T., Ducamp, C., Hatch, S. A., Blanchard, P., Danchin, E., & Wagner, R. H. (2014). Preen secretions encode information on MHC similarity in certain sex-dyads in a monogamous seabird. *Scientific Reports*, *4*, 6920. <https://doi.org/doi:10.1038/srep06920>

Lehnert, S. J., Helou, L., Pitcher, T. E., Heath, J. W., & Heath, D. D. (2018). Sperm competition, but not major histocompatibility divergence, drives differential fertilization success between alternative reproductive tactics in Chinook salmon. *Journal of Evolutionary Biology*, *31*(1), 88-97. <https://doi.org/10.1111/jeb.13199>

Lehnert, S. J., Pitcher, T. E., Devlin, R. H., & Heath, D. D. (2016). Red and white Chinook salmon: Genetic divergence and mate choice. *Molecular Ecology*, *25*(6), 1259-1274. <https://doi.org/10.1111/mec.13560>

Lei, W., Zhou, X., Fang, W., Lin, Q., & Chen, X. (2016). Major histocompatibility complex class II DAB alleles associated with intestinal parasite load in the vulnerable Chinese egret (Egretta eulophotes) [Article]. *Ecology and Evolution*, *6*(13), 4421-4434. <https://doi.org/10.1002/ece3.2226>

Lenz, T. L., Eizaguirre, C., Scharsack, J. P., Kalbe, M., & Milinski, M. (2009). Disentangling the role of MHC-dependent 'good genes' and 'compatible genes' in mate-choice decisions of three-spined sticklebacks Gasterosteus aculeatus under semi-natural conditions. *Journal of Fish Biology*, *75*(8), 2122-2142. <http://www.scopus.com/inward/record.url?eid=2-s2.0-72549089396&partnerID=40&md5=4141311ed7a3045c0e9b14667f6047ab>

Lenz, T. L., Hafer, N., Samonte, I. E., Yeates, S. E., & Milinski, M. (2018). Cryptic haplotype-specific gamete selection yields offspring with optimal MHC immune genes. *Evolution*, *72*(11), 2478-2490. <https://doi.org/10.1111/evo.13591>

Lenz, T. L., Mueller, B., Trillmich, F., & Wolf, J. B. (2013). Divergent allele advantage at MHC-DRB through direct and maternal genotypic effects and its consequences for allele pool composition and mating [Article]. *Proceedings. Biological sciences / The Royal Society*, *280*(1762), 20130714. <http://www.scopus.com/inward/record.url?eid=2-s2.0-84891543538&partnerID=40&md5=0077357eb42b381a467c1aab5ade3e42>

Lenz, T. L., Wells, K., Pfeiffer, M., & Sommer, S. (2009). Diverse MHC IIB allele repertoire increases parasite resistance and body condition in the long-tailed giant rat (Leopoldamys sabanus) [Article]. *BMC Evolutionary Biology*, *9*(1), Article 269. <https://doi.org/10.1186/1471-2148-9-269>

Li, C., Wang, X., Zhang, Q., Wang, Z., Qi, J., Yi, Q., Liu, Z., Wang, Y., & Yu, H. (2012). Identification of two major histocompatibility (MH) class II A genes and their association to Vibrio anguillarum infection in half-smooth tongue sole (Cynoglossus semilaevis) [Article]. *Journal of Ocean University of China*, *11*(1), 32-44. <https://doi.org/10.1007/s11802-012-1802-4>

Li, C., Zhang, Q., Yu, Y., Li, S., Zhong, Q., Sun, Y., Wang, Z., Qi, J., Zhai, J., & Wang, X. (2011). Sequence polymorphism of two major histocompatibility (MH) class II B genes and their association with Vibrio anguillarum infection in half-smooth tongue sole (Cynoglossus semilaevis) [Article]. *Chinese Journal of Oceanology and Limnology*, *29*(6), 1275-1286. <https://doi.org/10.1007/s00343-011-0278-1>

Li, L., Wang, B. B., Ge, Y. F., & Wan, Q. H. (2014). Major histocompatibility complex class II polymorphisms in forest musk deer (Moschus berezovskii) and their probable association with purulent disease [Article]. *International Journal of Immunogenetics*, *41*(5), 401-412. <https://doi.org/10.1111/iji.12135>

Liu, H. Y., He, K., Ge, Y. F., Wan, Q. H., & Fang, S. G. (2021). Cape feather coloration signals different genotypes of the most polymorphic mhc locus in male golden pheasants (Chrysolophus pictus). *Animals*, *11*(2), 1-7, Article 276. <https://doi.org/10.3390/ani11020276>

Liu, Z., Hu, D. D., Shao, S. J., Huang, J. Q., Wang, J. F., & Yang, J. (2013). Polymorphisms in major histocompatibility complex class IIα genes are associated with resistance to infectious hematopoietic necrosis in rainbow trout, *Oncorhynchus mykiss* (Walbaum, 1792) [Article]. *Journal of Applied Ichthyology*, *29*(6), 1234-1240. <https://doi.org/10.1111/jai.12326>

Lo, C. W., Takeshima, S. N., Wada, S., Matsumoto, Y., & Aida, Y. (2021). Bovine major histocompatibility complex (BoLA) heterozygote advantage against the outcome of bovine leukemia virus infection [Article]. *HLA*, *98*(2), 132-139. <https://doi.org/10.1111/tan.14285>

Loham, J., Grahm, M., Langefors, Å., Andersen, Ø., Storset, A., & Schantz, T. V. (2008). Experimental evidence for major histocompatibility complex-allele-specific resistance to a bacterial infection [Article]. *Hungarian Quarterly*, *49*(191), 2028-2033. <https://doi.org/10.1098/rspb.2002.2114>

Lohm, J., Grahn, M., Langefors, Å., Andersen, Ø., Storset, A., & Von Schantz, T. (2002). Experimental evidence for major histocompatibility complex-allele-specific resistance to a bacterial infection [Article]. *Proceedings of the Royal Society B: Biological Sciences*, *269*(1504), 2029-2033. <https://doi.org/10.1098/rspb.2002.2114>

Løvlie, H., Gillingham, M. A., Worley, K., Pizzari, T., & Richardson, D. S. (2013). Cryptic female choice favours sperm from major histocompatibility complex-dissimilar males [Article]. *Proceedings. Biological sciences / The Royal Society*, *280*(1769), 20131296. <http://www.scopus.com/inward/record.url?eid=2-s2.0-84897359944&partnerID=40&md5=f86a0235e8b9164ee3d7d4c07b593065>

MacManes, M. D., & Lacey, E. A. (2012). Is promiscuity associated with enhanced selection on MHC-DQα in mice (genus peromyscus)? *Plos One*, *7*(5), Article e37562. <https://doi.org/10.1371/journal.pone.0037562>

Matthews, B., Harmon, L. J., M'Gonigle, L., Marchinko, K. B., & Schaschl, H. (2010). Sympatric and allopatric divergence of MHC genes in threespine stickleback. *Plos One*, *5*(6), Article e10948. <https://doi.org/10.1371/journal.pone.0010948>

McCairns, R. J. S., Bourget, S., & Bernatchez, L. (2011). Putative causes and consequences of MHC variation within and between locally adapted stickleback demes [Article]. *Molecular Ecology*, *20*(3), 486-502. <https://doi.org/10.1111/j.1365-294X.2010.04950.x>

Meléndez-Rosa, J., Bi, K., & Lace, E. A. (2020). Mating system is correlated with immunogenetic diversity in sympatric species of Peromyscine mice. *Plos One*, *15*(7 July), Article e0236084. <https://doi.org/10.1371/journal.pone.0236084>

Meléndez-Rosa, J., Bi, K., & Lacey, E. A. (2018). Genomic analysis of MHC-based mate choice in the monogamous California mouse. *Behavioral Ecology*, *29*(5), 1167-1180. <https://doi.org/10.1093/beheco/ary096>

Meyer-Lucht, Y., & Sommer, S. (2009). Number of MHC alleles is related to parasite loads in natural populations of yellow necked mice, Apodemus flavicollis [Article]. *Evolutionary Ecology Research*, *11*(7), 1085-1097.

Milinski, M., Griffiths, S., Wegner, K. M., Reusch, T. B. H., Haas-Assenbaum, A., & Boehm, T. (2005). Mate choice decisions of stickleback females predictably modified by MHC peptide ligands. *Proceedings of the National Academy of Sciences of the United States of America*, *102*(12), 4414-4418. <http://www.scopus.com/inward/record.url?eid=2-s2.0-15444364324&partnerID=40&md5=52a921bc00097259e3c08234b1790b37>

Miller, K. M., Winton, J. R., Schulze, A. D., Purcell, M. K., & Ming, T. J. (2004). Major histocompatibility complex loci are associated with susceptibility of Atlantic salmon to infectious hematopoietic necrosis virus [Conference Paper]. *Environmental Biology of Fishes*, *69*(1-4), 307-316. <https://doi.org/10.1023/B:EBFI.0000022874.48341.0f>

Mingju, E., Song, X., Wang, L., Yang, Y., Wei, X., Yu, J., Gong, Y., & Wang, H. (2021). Mate choice for major histocompatibility complex (MHC) complementarity in the Yellow-rumped Flycatcher (Ficedula zanthopygia). *Avian Research*, *12*(1), Article 27. <https://doi.org/10.1186/s40657-021-00261-w>

Minias, P., Gómez, J., & Wlodarczyk, R. (2020). Egg spottiness reflects female condition, physiological stress, and ornament expression in a common rallid species. *Auk*, *137*(4), Article ukaa054. <https://doi.org/10.1093/auk/ukaa054>

Molee, A., Kongroi, K., Kuadsantia, P., Poompramun, C., & Likitdecharote, B. (2016). Association between single nucleotide polymorphisms of the major histocompatibility complex class II gene and Newcastle disease virus Titre and body weight in Leung Hang Khao chickens [Article]. *Asian-Australasian Journal of Animal Sciences*, *29*(1), 29-35. <https://doi.org/10.5713/ajas.15.0029>

Montero, B. K., Uddin, W., Schwensow, N., Gillingham, M. A. F., Ratovonamana, Y. R., Rakotondranary, S. J., Corman, V., Drosten, C., Ganzhorn, J. U., & Sommer, S. (2021). Evidence of MHC class I and II influencing viral and helminth infection via the microbiome in a non-human primate [Article]. *PLoS Pathogens*, *17*(11), Article e1009675. <https://doi.org/10.1371/journal.ppat.1009675>

Natsopoulou, M. E., Pálsson, S., & Ólafsdóttir, G. A. (2012). Parasites and parallel divergence of the number of individual MHC alleles between sympatric three-spined stickleback Gasterosteus aculeatus morphs in Iceland [Article]. *Journal of Fish Biology*, *81*(5), 1696-1714. <https://doi.org/10.1111/j.1095-8649.2012.03430.x>

Neff, B. D., Garner, S. R., Heath, J. W., & Heath, D. D. (2008). The MHC and non-random mating in a captive population of Chinook salmon. *Heredity*, *101*(2), 175-185. <http://www.scopus.com/inward/record.url?eid=2-s2.0-47949108438&partnerID=40&md5=032b94eac25fcc9e2a40b8e8d2521d87>

Oliver, M. K., Telfer, S., & Piertney, S. B. (2009). Major histocompatibility complex (MHC) heterozygote superiority to natural multi-parasite infections in the water vole (Arvicola terrestris). *Proceedings of the Royal Society B: Biological Sciences*, *276*(1659), 1119-1128. <http://www.scopus.com/inward/record.url?eid=2-s2.0-60049096475&partnerID=40&md5=da7fcbd4527b64c083abbdb85f8dd7ca>

Osborne, A. J., Pearson, J., Negro, S. S., Louise Chilvers, B., Kennedy, M. A., & Gemmell, N. J. (2015). Heterozygote advantage at MHC DRB may influence response to infectious disease epizootics. *Molecular Ecology*, *24*(7), 1419-1432. <https://doi.org/10.1111/mec.13128>

Osborne, M. J., Pilger, T. J., Lusk, J. D., & Turner, T. F. (2017). Spatio-temporal variation in parasite communities maintains diversity at the major histocompatibility complex class IIβ in the endangered Rio Grande silvery minnow. *Molecular Ecology*, *26*(2), 471-489. <https://doi.org/10.1111/mec.13936>

Ottová, E., Šimková, A., & Morand, S. (2007). The role of major histocompatibility complex diversity in vigour of fish males (Abramis brama L.) and parasite selection. *Biological Journal of the Linnean Society*, *90*(3), 525-538. <https://doi.org/10.1111/j.1095-8312.2007.00743.x>

Pearson, S. K., Bull, C. M., & Gardner, M. G. (2018). Selection outweighs drift at a fine scale: Lack of MHC differentiation within a family living lizard across geographically close but disconnected rocky outcrops. *Molecular Ecology*, *27*(9), 2204-2214. <https://doi.org/10.1111/mec.14571>

Pearson, S. K., Godfrey, S. S., Schwensow, N., Bull, C. M., & Gardner, M. G. (2017). Genes and group membership predict Gidgee skink (Egernia stokesii) reproductive pairs. *Journal of Heredity*, *108*(4), 369-378. <https://doi.org/10.1093/jhered/esx026>

Pitcher, T. E., & Neff, B. D. (2007). Genetic quality and offspring performance in Chinook salmon: Implications for supportive breeding. *Conservation Genetics*, *8*(3), 607-616. <https://doi.org/10.1007/s10592-006-9204-z>

Promerová, M., Alavioon, G., Tusso, S., Burri, R., & Immler, S. (2017). No evidence for MHC class II-based non-random mating at the gametic haplotype in Atlantic salmon. *Heredity*, *118*(6), 563-567. <https://doi.org/10.1038/hdy.2016.129>

Promerová, M., Králová, T., Bryjová, A., Albrecht, T., & Bryja, J. (2013). MHC Class IIB Exon 2 Polymorphism in the Grey Partridge (Perdix perdix) Is Shaped by Selection, Recombination and Gene Conversion. *Plos One*, *8*(7), Article e69135. <https://doi.org/10.1371/journal.pone.0069135>

Radwan, J., Demiaszkiewicz, A. W., Kowalczyk, R., Lachowicz, J., Kawałko, A., Wójcik, J. M., Pyziel, A. M., & Babik, W. (2010). An evaluation of two potential risk factors, MHC diversity and host density, for infection by an invasive nematode Ashworthius sidemi in endangered European bison (Bison bonasus) [Article]. *Biological Conservation*, *143*(9), 2049-2053. <https://doi.org/10.1016/j.biocon.2010.05.012>

Radwan, J., Tkacz, A., & Kloch, A. (2008). MHC and preferences for male odour in the bank vole [Article]. *Ethology*, *114*(9), 827-833. <https://doi.org/10.1111/j.1439-0310.2008.01528.x>

Rakus, K. Ł., Wiegertjes, G. F., Jurecka, P., Walker, P. D., Pilarczyk, A., & Irnazarow, I. (2009). Major histocompatibility (MH) class II B gene polymorphism influences disease resistance of common carp (Cyprinus carpio L.) [Article]. *Aquaculture*, *288*(1-2), 44-50. <https://doi.org/10.1016/j.aquaculture.2008.11.016>

Rauch, G., Kalbe, M., & Reusch, T. B. H. (2006). Relative importance of MHC and genetic background for parasite load in a field experiment [Article]. *Evolutionary Ecology Research*, *8*(2), 373-386.

Reichard, M., Spence, R., Bryjová, A., Bryja, J., & Smith, C. (2012). Female rose bitterling prefer MHC-dissimilar males: Experimental evidence. *Plos One*, *7*(7), Article e40780. <https://doi.org/10.1371/journal.pone.0040780>

Rekdal, S. L., Anmarkrud, J. A., Lifjeld, J. T., & Johnsen, A. (2019). Extra-pair mating in a passerine bird with highly duplicated major histocompatibility complex class II: Preference for the golden mean. *Molecular Ecology*, *28*(23), 5133-5144. <https://doi.org/10.1111/mec.15273>

Rekdal, S. L., Anmarkrud, J. A., Lifjeld, J. T., & Johnsen, A. (2021). Elevated phytohaemagglutinin-induced skin-swelling response at an intermediate number of MHC class II alleles in bluethroat nestlings. *Journal of Avian Biology*, *52*(5), Article e02734. <https://doi.org/10.1111/jav.02734>

Reusch, T. B. H., Häberli, M. A., Aeschlimann, P. B., & Milinski, M. (2001). Female sticklebacks count alleles in a strategy of sexual selection explaining MHC polymorphism [Article]. *Nature*, *414*(6861), 300-302. <https://doi.org/10.1038/35104547>

Roberts, S. C., Hale, M. L., & Petrie, M. (2006). Correlations between heterozygosity and measures of genetic similarity: implications for understanding mate choice. *Journal of Evolutionary Biology*, *19*(2), 558-569. <Go to ISI>://000235985400027

Rymešová, D., Králová, T., Promerová, M., Bryja, J., Tomášek, O., Svobodová, J., Šmilauer, P., Šálek, M., & Albrecht, T. (2017). Mate choice for major histocompatibility complex complementarity in a strictly monogamous bird, the grey partridge (Perdix perdix). *Frontiers in Zoology*, *14*(1), Article 9. <https://doi.org/10.1186/s12983-017-0194-0>

Santos, P. S. C., Courtiol, A., Heidel, A. J., Höner, O. P., Heckmann, I., Nagy, M., Mayer, F., Platzer, M., Voigt, C. C., & Sommer, S. (2016). MHC-dependent mate choice is linked to a trace-amine-associated receptor gene in a mammal. *Scientific Reports*, *6*, Article 38490. <https://doi.org/10.1038/srep38490>

Santos, P. S. C., Michler, F. U., & Sommer, S. (2017). Can MHC-assortative partner choice promote offspring diversity? A new combination of MHC-dependent behaviours among sexes in a highly successful invasive mammal. *Molecular Ecology*, *26*(8), 2392-2404. <https://doi.org/10.1111/mec.14035>

Sauermann, U., Nürnberg, P., Bercovitch, F. B., Berard, J. D., Trefilov, A., Widdig, A., Kessler, M., Schmidtke, J., & Krawczak, M. (2001). Increased reproductive success of MHC class II heterozygous males among free-ranging rhesus macaques. *Human Genetics*, *108*(3), 249-254. <https://doi.org/10.1007/s004390100485>

Savage, A. E., Muletz-Wolz, C. R., Campbell Grant, E. H., Fleischer, R. C., & Mulder, K. P. (2019). Functional variation at an expressed MHC class IIβ locus associates with Ranavirus infection intensity in larval anuran populations. *Immunogenetics*, *71*(4), 335-346. <https://doi.org/10.1007/s00251-019-01104-1>

Savage, A. E., & Zamudio, K. R. (2016). Adaptive tolerance to a pathogenic fungus drives major histocompatibility complex evolution in natural amphibian populations. *Proceedings of the Royal Society B: Biological Sciences*, *283*(1827), Article 20153115. <https://doi.org/10.1098/rspb.2015.3115>

Sawada, A., Ando, H., & Takagi, M. (2020). Evaluating the existence and benefit of major histocompatibility complex-based mate choice in an isolated owl population. *Journal of Evolutionary Biology*, *33*(6), 762-772. <https://doi.org/10.1111/jeb.13629>

Schad, J., Dechmann, D. K. N., Voigt, C. C., & Sommer, S. (2011). MHC class II DRB diversity, selection pattern and population structure in a neotropical bat species, Noctilio albiventris. *Heredity*, *107*(2), 115-126. <https://doi.org/10.1038/hdy.2010.173>

Schad, J., Ganzhorn, J. U., & Sommer, S. (2005). Parasite burden and constitution of major histocompatibility complex in the Malagasy mouse lemur, *Microcebus murinus*. *Evolution*, *59*(2), 439-450. <http://www.scopus.com/inward/record.url?eid=2-s2.0-14844365794&partnerID=40&md5=f37037e81a67b615ac467e4ebda07095>

Schaschl, H., Tobler, M., Plath, M., Penn, D. J., & Schlupp, I. (2008). Polymorphic MHC loci in an asexual fish, the amazon molly (Poecilia formosa; Poeciliidae). *Molecular Ecology*, *17*(24), 5220-5230. <https://doi.org/10.1111/j.1365-294X.2008.03997.x>

Schwensow, N., Dausmann, K., Eberle, M., Fietz, J., & Sommer, S. (2010). Functional associations of similar MHC alleles and shared parasite species in two sympatric lemurs [Article]. *Infection, Genetics and Evolution*, *10*(5), 662-668. <https://doi.org/10.1016/j.meegid.2010.03.012>

Schwensow, N., Eberle, M., & Sommer, S. (2008). Compatibility counts: MHC-associated mate choice in a wild promiscuous primate. *Proceedings of the Royal Society B: Biological Sciences*, *275*(1634), 555-564. <http://www.scopus.com/inward/record.url?eid=2-s2.0-38549167904&partnerID=40&md5=8c673186d722f75156803b8f4bbce834>

Schwensow, N., Eberle, M., & Sommer, S. (2010). Are there ubiquitous parasite-driven major histocompatibility complex selection mechanisms in gray mouse lemurs? [Article]. *International Journal of Primatology*, *31*(4), 519-537. <https://doi.org/10.1007/s10764-010-9411-9>

Schwensow, N., Fietz, J., Dausmann, K., & Sommer, S. (2008). MHC-associated mating strategies and the importance of overall genetic diversity in an obligate pair-living primate. *Evolutionary Ecology*, *22*(5), 617-636. <http://www.scopus.com/inward/record.url?eid=2-s2.0-50249138364&partnerID=40&md5=b39afa5cda5871da996c0e1cf7a8782e>

Schwensow, N., Fietz, J., Dausmann, K. H., & Sommer, S. (2007). Neutral versus adaptive genetic variation in parasite resistance: Importance of major histocompatibility complex supertypes in a free-ranging primate [Article]. *Heredity*, *99*(3), 265-277. <https://doi.org/10.1038/sj.hdy.6800993>

Seifertová, M., Jarkovský, J., & Šimková, A. (2016). Does the parasite-mediated selection drive the MHC class IIB diversity in wild populations of European chub (Squalius cephalus)? [Article]. *Parasitology Research*, *115*(4), 1401-1415. <https://doi.org/10.1007/s00436-015-4874-4>

Setchell, J. M., Abbott, K. M., Gonzalez, J. P., & Knapp, L. A. (2013). Testing for post-copulatory selection for major histocompatibility complex genotype in a semi-free-ranging primate population. *American Journal of Primatology*, *75*(10), 1021-1031. <https://doi.org/10.1002/ajp.22166>

Setchell, J. M., Charpentier, M. J. E., Abbott, K. M., Wickings, E. J., & Knapp, L. A. (2009). Is brightest best? Testing the Hamilton-Zuk hypothesis in Mandrills. *International Journal of Primatology*, *30*(6), 825-844. <https://doi.org/10.1007/s10764-009-9371-0>

Setchell, J. M., Charpentier, M. J. E., Abbott, K. M., Wickings, E. J., & Knapp, L. A. (2010). Opposites attract: MHC-associated mate choice in a polygynous primate. *Journal of Evolutionary Biology*, *23*(1), 136-148. <http://www.scopus.com/inward/record.url?eid=2-s2.0-72449182780&partnerID=40&md5=c1bea84ea288cbc0fc2980b3d7e02103>

Silveira, L., Garner, S. R., & Neff, B. D. (2020). Similarity at the major histocompatibility complex class II does not influence mating patterns in bluegill (Lepomis macrochirus). *Behavioral Ecology and Sociobiology*, *74*(3), Article 38. <https://doi.org/10.1007/s00265-020-2822-5>

Šimková, A., Ottová, E., & Morand, S. (2006). MHC variability, life-traits and parasite diversity of European cyprinid fish [Article]. *Evolutionary Ecology*, *20*(5), 465-477. <https://doi.org/10.1007/s10682-006-0014-z>

Sin, Y. W., Annavi, G., Dugdale, H. L., Newman, C., Burke, T., & MacDonald, D. W. (2014). Pathogen burden, co-infection and major histocompatibility complex variability in the European badger (Meles meles). *Molecular Ecology*, *23*(20), 5072-5088. <https://doi.org/10.1111/mec.12917>

Sin, Y. W., Annavi, G., Newman, C., Buesching, C., Burke, T., Macdonald, D. W., & Dugdale, H. L. (2015). MHC class II-assortative mate choice in European badgers (Meles meles). *Molecular Ecology*, *24*(12), 3138-3150. <https://doi.org/10.1111/mec.13217>

Singh, P. K., Singh, S. V., Singh, M. K., Saxena, V. K., Horin, P., Singh, A. V., & Sohal, J. S. (2012). Effect of genetic variation in the MHC class II DRB region on resistance and susceptibility to Johne's disease in endangered Indian Jamunapari goats. *International Journal of Immunogenetics*, *39*(4), 314-320. <https://doi.org/10.1111/j.1744-313X.2012.01092.x>

Skarstein, F., Folstad, I., Liljedal, S., & Grahn, M. (2005). MHC and fertilization success in the Arctic charr (Salvelinus alpinus). *Behavioral Ecology and Sociobiology*, *57*(4), 374-380. <https://doi.org/10.1007/s00265-004-0860-z>

Slade, J. W. G., Sarquis-Adamson, Y., Gloor, G. B., Lachance, M. A., & MacDougall-Shackleton, E. A. (2017). Population differences at MHC do not explain enhanced resistance of song sparrows to local parasites. *Journal of Heredity*, *108*(2), 127-134. <https://doi.org/10.1093/jhered/esw082>

Slade, J. W. G., Watson, M. J., Kelly, T. R., Gloor, G. B., Bernards, M. A., & Macdougall-Shackleton, E. A. (2016). Chemical composition of preen wax reflects major histocompatibility complex similarity in songbirds. *Proceedings of the Royal Society B: Biological Sciences*, *283*(1842), Article 20161966. <https://doi.org/10.1098/rspb.2016.1966>

Slade, J. W. G., Watson, M. J., & MacDougall-Shackleton, E. A. (2019). “Balancing” balancing selection? Assortative mating at the major histocompatibility complex despite molecular signatures of balancing selection. *Ecology and Evolution*, *9*(9), 5146-5157. <https://doi.org/10.1002/ece3.5087>

Smith, C., Ondračková, M., Spence, R., Adams, S., Betts, D. S., & Mallon, E. (2011). Pathogen-mediated selection for MHC variability in wild zebrafish [Article]. *Evolutionary Ecology Research*, *13*(6), 589-605.

Smith, C., Spence, R., & Reichard, M. (2018). Sperm is a sexual ornament in rose bitterling. *Journal of Evolutionary Biology*, *31*(11), 1610-1622. <https://doi.org/10.1111/jeb.13357>

Sommer, S. (2005). Major histocompatibility complex and mate choice in a monogamous rodent [Article]. *Behavioral Ecology and Sociobiology*, *58*(2), 181-189. <https://doi.org/10.1007/s00265-005-0909-7>

Sommer, S., Schwab, D., & Ganzhorn, J. U. (2002). MHC diversity of endemic Malagasy rodents in relation to geographic range and social system. *Behavioral Ecology and Sociobiology*, *51*(3), 214-221. <https://doi.org/10.1007/s00265-001-0432-4>

Soutter, F., Martorell, S., Solano-Gallego, L., & Catchpole, B. (2018). Inconsistent MHC class II association in Beagles experimentally infected with Leishmania infantum [Article]. *Veterinary Journal*, *235*, 9-15. <https://doi.org/10.1016/j.tvjl.2018.03.001>

Stear, A., Ali, A. O. A., Brujeni, G. N., Buitkamp, J., Donskow-Łysoniewska, K., Fairlie-Clarke, K., Groth, D., Isa, N. M. M., & Stear, M. J. (2019). Identification of the amino acids in the Major Histocompatibility Complex class II region of Scottish Blackface sheep that are associated with resistance to nematode infection [Article]. *International Journal for Parasitology*, *49*(10), 797-804. <https://doi.org/10.1016/j.ijpara.2019.05.003>

Sterck, E. H. M., Bontrop, R. E., de Groot, N., de Vos-Rouweler, A. J. M., & Doxiadis, G. G. M. (2017). No postcopulatory selection against MHC-homozygous offspring: Evidence from a pedigreed captive rhesus macaque colony. *Molecular Ecology*, *26*(14), 3785-3793. <https://doi.org/10.1111/mec.14153>

Strandh, M., Westerdahl, H., Pontarp, M., Canbäck, B., Dubois, M. P., Miquel, C., Taberlet, P., & Bonadonna, F. (2012). Major histocompatibility complex class II compatibility, but not class I, predicts mate choice in a bird with highly developed olfaction. *Proceedings of the Royal Society B: Biological Sciences*, *279*(1746), 4457-4463. <http://www.scopus.com/inward/record.url?eid=2-s2.0-84866842100&partnerID=40&md5=274db3772ba1bc823aec67ba95b33309>

Sun, L., Zhou, T., Stone, G. N., Wan, Q. H., & Fang, S. G. (2019). Seeing-good-gene-based mate choice: From genes to behavioural preferences. *Journal of Animal Ecology*, *88*(11), 1708-1719. <https://doi.org/10.1111/1365-2656.13071>

Tentelier, C., Barroso-Gomila, O., Lepais, O., Manicki, A., Romero-Garmendia, I., & Jugo, B. M. (2017). Testing mate choice and overdominance at MH in natural families of Atlantic salmon Salmo salar. *Journal of Fish Biology*, *90*(4), 1644-1659. <https://doi.org/10.1111/jfb.13260>

Thoß, M., Ilmonen, P., Musolf, K., & Penn, D. J. (2011). Major histocompatibility complex heterozygosity enhances reproductive success. *Molecular Ecology*, *20*(7), 1546-1557. <http://www.scopus.com/inward/record.url?eid=2-s2.0-79953047741&partnerID=40&md5=fa40e0a2f2af2e40c8e9efe6c386f877>

Tollenaere, C., Bryja, J., Galan, M., Cadet, P., Deter, J., Chaval, Y., Berthier, K., Ribas Salvador, A., Voutilainen, L., Laakkonen, J., Henttonen, H., Cosson, J. F., & Charbonnel, N. (2008). Multiple parasites mediate balancing selection at two MHC class II genes in the fossorial water vole: Insights from multivariate analyses and population genetics [Article]. *Journal of Evolutionary Biology*, *21*(5), 1307-1320. <https://doi.org/10.1111/j.1420-9101.2008.01563.x>

Trujillo, A. L., Hoffman, E. A., Becker, C. G., & Savage, A. E. (2021). Spatiotemporal adaptive evolution of an MHC immune gene in a frog-fungus disease system [Article]. *Heredity*, *126*(4), 640-655. <https://doi.org/10.1038/s41437-020-00402-9>

Turner, S. M., Chaves-Campos, J., & DeWoody, J. A. (2009). Parental relatedness and major histocompatibility effects on early embryo survivorship in Atlantic salmon. *Genetica*, *137*(1), 99-109. <https://doi.org/10.1007/s10709-009-9354-2>

Wedekind, C., Walker, M., Portmann, J., Cenni, B., Müller, R., & Binz, T. (2004). MHC-linked susceptibility to a bacterial infection, but no MHC-linked cryptic female choice in whitefish [Article]. *Journal of Evolutionary Biology*, *17*(1), 11-18. <https://doi.org/10.1046/j.1420-9101.2004.00669.x>

Wegner, K. M., Kalbe, M., Milinski, M., & Reusch, T. B. H. (2008). Mortality selection during the 2003 European heat wave in three-spined sticklebacks: Effects of parasites and MHC genotype [Article]. *BMC Evolutionary Biology*, *8*(1), Article 124. <https://doi.org/10.1186/1471-2148-8-124>

Wegner, K. M., Reusch, T. B. H., & Kalbe, M. (2003). Multiple parasites are driving major histocompatibility complex polymorphism in the wild [Article]. *Journal of Evolutionary Biology*, *16*(2), 224-232. <https://doi.org/10.1046/j.1420-9101.2003.00519.x>

Weir, L. K., Hutchings, J. A., & Heath, D. D. (2012). Influence of density and major histocompatibility genotype on sexual selection in a salmonid alternative mating strategy. *Canadian Journal of Fisheries and Aquatic Sciences*, *69*(4), 670-680. <https://doi.org/10.1139/F2012-007>

Whittingham, L. A., Dunn, P. O., Freeman-Gallant, C. R., Taff, C. C., & Johnson, J. A. (2018). Major histocompatibility complex variation and blood parasites in resident and migratory populations of the common yellowthroat. *Journal of Evolutionary Biology*, *31*(10), 1544-1557. <https://doi.org/10.1111/jeb.13349>

Whittingham, L. A., Freeman-Gallant, C. R., Taff, C. C., & Dunn, P. O. (2015). Different ornaments signal male health and MHC variation in two populations of a warbler. *Molecular Ecology*, *24*(7), 1584-1595. <https://doi.org/10.1111/mec.13130>

Widdig, A., Bercovitch, F. B., Streich, W. J., Sauermann, U., Nürnberg, P., & Krawczak, M. (2004). A longitudinal analysis of reproductive skew in male rhesus macaques. *Proceedings of the Royal Society B: Biological Sciences*, *271*(1541), 819-826. <https://doi.org/10.1098/rspb.2003.2666>

Winternitz, J. C., Wares, J. P., Yabsley, M. J., & Altizer, S. (2014). Wild cyclic voles maintain high neutral and MHC diversity without strong evidence for parasite-mediated selection. *Evolutionary Ecology*, *28*(5), 957-975. <https://doi.org/10.1007/s10682-014-9709-8>

Wynne, J. W., Cook, M. T., Nowak, B. F., & Elliott, N. G. (2007). Major histocompatibility polymorphism associated with resistance towards amoebic gill disease in Atlantic salmon (Salmo salar L.) [Article]. *Fish and Shellfish Immunology*, *22*(6), 707-717. <https://doi.org/10.1016/j.fsi.2006.08.019>

Xu, T. j., Chen, S. l., Ji, X. s., & Tian, Y. s. (2008). MHC polymorphism and disease resistance to Vibrio anguillarum in 12 selective Japanese flounder (Paralichthys olivaceus) families. *Fish and Shellfish Immunology*, *25*(3), 213-221. <https://doi.org/10.1016/j.fsi.2008.05.007>

Yang, B., Ren, B., Xiang, Z., Yang, J., Yao, H., Garber, P. A., & Li, M. (2014). Major histocompatibility complex and mate choice in the polygynous primate: The Sichuan snub-nosed monkey (Rhinopithecus roxellana). *Integrative Zoology*, *9*(5), 598-612. <https://doi.org/10.1111/1749-4877.12084>

Yang, J., Liu, Z., Shi, H. N., Zhang, J. P., Wang, J. F., Huang, J. Q., & Kang, Y. J. (2016). Association between MHC II beta chain gene polymorphisms and resistance to infectious haematopoietic necrosis virus in rainbow trout (Oncorhynchus mykiss, Walbaum, 1792) [Article]. *Aquaculture Research*, *47*(2), 570-578. <https://doi.org/10.1111/are.12516>

Yang, M., Wei, J., Li, P., Wei, S., Huang, Y., & Qin, Q. (2016). MHC polymorphism and disease resistance to Singapore grouper iridovirus (SGIV) in the orange-spotted grouper, Epinephelus coioides [Article]. *Science Bulletin*, *61*(9), 693-699. <https://doi.org/10.1007/s11434-016-1055-5>

Yu, L., Nie, Y., Yan, L., Hu, Y., & Wei, F. (2018). No evidence for MHC-based mate choice in wild giant pandas. *Ecology and Evolution*, *8*(17), 8642-8651. <https://doi.org/10.1002/ece3.4419>

Yu, X., Zheng, R., Zhang, J., Shen, B., & Dong, B. (2014). Genetic polymorphism of major histocompatibility complex class IIB alleles and pathogen resistance in the giant spiny frog Quasipaa spinosa. *Infection, Genetics and Evolution*, *28*, 175-182. <https://doi.org/10.1016/j.meegid.2014.09.028>

Zhang, B. Y., Hu, H. Y., Song, C. M., Huang, K., Dunn, D. W., Yang, X., Wang, X. W., Zhao, H. T., Wang, C. L., Zhang, P., & Li, B. G. (2020). MHC-Based Mate Choice in Wild Golden Snub-Nosed Monkeys. *Frontiers in Genetics*, *11*, Article 609414. <https://doi.org/10.3389/fgene.2020.609414>

Zhang, L., Wu, Q., Hu, Y., Wu, H., & Wei, F. (2015). Major histocompatibility complex alleles associated with parasite susceptibility in wild giant pandas. *Heredity*, *114*(1), 85-93. <https://doi.org/10.1038/hdy.2014.73>

Zhang, M., & He, H. (2013). Parasite-mediated selection of major histocompatibility complex variability in wild brandt's voles (Lasiopodomys brandtii) from Inner Mongolia, China. *BMC Evolutionary Biology*, *13*(1), Article 149. <https://doi.org/10.1186/1471-2148-13-149>

Zhu, B., Zhu, Z., Wang, J., Huang, S., Li, F., Wang, L., Liu, Y., Yan, Q., Zhou, S., Lu, M., Yang, D., & Wang, B. (2018). Chinese woodchucks with different susceptibility to WHV infection differ in their genetic background exemplified by cytochrome B and MHC-DRB molecules. *Virology Journal*, *15*(1), Article 101. <https://doi.org/10.1186/s12985-018-1010-y>
